# *Akkermansia muciniphila* inhibits tryptophan metabolism via the AhR/β-catenin signaling pathway to counter the progression of colorectal cancer

**DOI:** 10.7150/ijbs.85712

**Published:** 2023-08-21

**Authors:** Lu Zhang, Qing Ji, Qian Chen, Zhenzhen Wei, Shuochuan Liu, Long Zhang, Yuli Zhang, Zan Li, Huaimin Liu, Hua Sui

**Affiliations:** 1 Department of Combine Traditional Chinese & Western, The Affiliated Cancer Hospital of Zhengzhou University & Henan Cancer Hospital, Zhengzhou, 450008, China.; 2 Department of Medical Oncology and Cancer Institute, Shuguang Hospital, Shanghai University of Traditional Chinese Medicine, Shanghai 201203, China.; 3 Department of critical care medicine, Henan Provincial Hospital of Traditional Chinese Medicine, Zhengzhou, 450002, China.; 4 Medical Experiment Center, Jiading Branch of Shanghai General Hospital, Shanghai Jiao Tong University School of Medicine, Shanghai 201803, China.; 5 Shanghai General Hospital Jiading Branch-Pharmacy school of Shanghai University of Traditional Chinese Medicine Joint Laboratory, Translational medicine Research Center for Cancer Prevention and Treatment, Shanghai 201803, China.; 6 Department of Breast disease, Henan Breast Cancer Center, Affiliated Cancer Hospital of Zhengzhou University & Henan Cancer Hospital, Zhengzhou, 450008, China.; 7 University of Shanghai for Science and Technology and Shanghai Changzheng Hospital Joint Research Center for Orthopedic Oncology, Institute of Biomedical Sciences and Clinical Technology Transformation, School of Health Science and Engineering, University of Shanghai for Science and Technology, Shanghai 200093, China.

**Keywords:** *Akkermansia muciniphila*, Tryptophan metabolism, AhR, Wnt/β-catenin signaling, Tumorigenesis

## Abstract

*Akkermansia muciniphila* (*A. muciniphila*), a gram-negative anaerobic bacterium, is selectively decreased in the fecal microbiota of patients with colorectal cancer (CRC), but its molecular mechanism in CRC development remains inconclusive. In this study, we first confirmed the inhibitory effect of *A. muciniphila* on CRC formation and analyzed the metabolic role of intestinal flora in human Polyps, A-CRA (advanced colorectal adenoma) and CRC samples. To better clarify the role of *A. muciniphila* in CRC development, a pseudo-germ-free (GF) azoxymethane (AOM)/dextran sulfate sodium (DSS) mouse model was established, followed by infection with or without *A. muciniphila*. Metabolomic analysis and RNA-seq analysis showed tryptophan-mediated aryl hydrocarbon receptor (AhR) was significantly down-regulated in *A. muciniphila*-infected CRC mice. Then, mice with intestinal specific AhR deficiency (AhR^fl/fl^ Cre) were generated and were used in 2 murine models: AOM/DSS treatment as a model of carcinogen-induced colon cancer and a genetically induced model using *Apc^Min/+^* mice. Notably, AhR deficiency inhibited CRC growth in the AOM/DSS and *Apc^Min/+^* mouse model. Moreover, AhR deficiency inhibited, rather than enhanced, tumor formation and tumor-derived organoids in Apc-deficient cells both *in vivo* and *in vitro* by activating Wnt/β-catenin signaling and TCF4/LEF1-dependent transcription. Furthermore, the antitumor effectiveness of *A. muciniphila* was abolished either in a human colon cancer tumor model induced by subcutaneous transplantation of AhR-silenced CRC cells, or AhR-deficienty spontaneous colorectal cancer model. In conclusion, supplementation with *A. muciniphila*. protected mice from CRC development by specifically inhibiting tryptophan-mediated AhR/β-catenin signaling.

## Introduction

Colorectal cancer (CRC), a most common cancer, brings about nearly 1,000,000 new cases and more than 600,000 deaths annually worldwide 1. One early benchmark of CRC is the formation of aberrant crypt foci (ACF) 2. Unlike many other cancers that develop in a relatively sterile environment, CRC occurs at the largest interface between host and gut microbial communities, and often involves inflammatory responses 3. Many recent studies have reported the genotoxicity of species associated with CRC, including colibactin-producing polyketide synthase *(pks)^+^ E. coli* 4, enterotoxigenic *B. fragilis* (ETBF) 5, *E. faecalis* 6 and cytolethal distending toxin-producing *Campylobacter jejuni* 7. However, their beneficial effects have been neglected.

*Akkermansia muciniphila* (*A. muciniphila*), a gram-negative anaerobic bacterium and the only representative member of the *Verrucomicrobia* phylum in the human intestinal tract, has been considered as a promising probiotic 8. Clinical data have shown that *A. muciniphila* is significantly enriched in the fecal and mucosal samples from healthy people, compared with that from CRC patients 9. Moreover, the high abundance of *A. muciniphila* is associated with a better clinical outcome 10. Our previous study has showed a significantly lower abundance of *A. muciniphila* in *Apc^Min/+^* mice than in wide type mice 11. Consistent with our study, *A. muciniphila* can also be reported to suppresse colorectal tumorigenesis in *Apc^Min/+^* mice by targeting TAMs in the tumor immune microenvironment 12. Specifically, *A. muciniphila* activates TLR2/NF-κB and NLRP3, leading to increased M1-like TAMs and the suppression of colonic tumorigenesis 12. In immunodeficient settings, however, *A. muciniphila* may act as a pathobiont to promote colitis in a genetically susceptible host 13,14. The molecular mechanism of *A. muciniphila* in countering human CRC remains inconclusive.

*A. muciniphila* can regulate cancer cell growth by directly triggering mucin degradation-related gene expression 15. However, little is known about how *A. muciniphila* inhibits inflammation, repairs the intestinal structure, and interacts with beneficial bacteria in the intestinal tract. Intestinal microbiota can regulate inflammatory response, chemotherapy resistance, and prognosis of carcinomatosis 16-18. Host-microbiota maladaptation in the inflammatory pathogenesis of CRC has aroused research interest.

The intestinal microbiota modulates the metabolism of tryptophan (Trp) during the host immunity. As an essential nutrient in mammals, Trp and its endogenous metabolites help maintain gut immune homeostasis in several immune diseases 19. The aryl hydrocarbon receptor (AhR) is activated by Trp catabolites to enhance tumor malignancy and suppress anti-tumor immunity 20,21. AhR, also known as the dioxin receptor, is a member of the basic helix-loop-helix/Per-AhR nuclear trans-locator-Sim homology superfamily. It mediates a wide variety of pharmacological and toxicological activities, such as drug-metabolizing enzyme induction, tumor promotion, teratogenesis, immunosuppression and wasting syndrome 22. *A. muciniphila* can restore AhR ligands in sucralose-consuming mice, consequently ameliorating nonalcoholic fatty liver disease 23. However, whether microbial dysbiosis impacts on AhR-driven mucosal reactivity, and whether AhR can be activated by microbiota-derived metabolites have not been answered.

Kynurenine (Kyn) pathway can be initiated by indoleamine-2,3-dioxygenase 1/2 (IDO1/2) or tryptophan-2,3-dioxgenase (TDO2) to regulate Trp catabolism in humans, yielding AhR agonists Kyn and kynurenic acid (KynA). AhR suppresses rather than promotes the development of glioblastoma 24. Several Trp metabolites can inactivate Wnt/β-catenin in esophageal cancer, indicating the inhibitory role of Wnt/β-catenin in cancers 25.

## Materials and methods

### Cell culture and reagents

Human colorectal DLD-1 cells and normal colon epithelial NCM460 cells were purchased from the the Cell Bank of Type Culture Collection of Chinese Academy of Sciences (Shanghai, China), and cultured in McCoy's 5A and DMEM medium respectively, both supplemented with 10% fetal bovine serum (Gibco, NY, USA), 2 mM glutamine, 100 units/ml streptomycin and penicillin (Invitrogen, Carlsbad, CA). The cells were grown at 37°C in a humidified 5% CO_2_ atmosphere. Monoclonal antibodies specific for Ki67 (ab1667), PCNA (ab92552), AhR (83200S), β-catenin (8480S), LEF1 (2230S), TCF4 (2569S), GAPDH (5174S) and β-actin (ab179467) were obtained from Abcam plc. (Cambridge, UK) and Cell Signaling Technology (Beverly, MA, USA).

### Tissue sampling

Polyp, A-CRA (advanced colorectal adenoma) and CRC tissue samples were obtained from 126 patients (cohort 1) having undergone resection of polyps (n = 46), adenomas (n = 38) and CRC (n = 42). According to Mayo endoscopic score, A-CRA was defined as the CRA keeping active for more than 6 months. The clinical study protocol conformed to the ethical guidelines of the 1975 Declaration of Helsinki and was approved by the institutional review boards.

### A. muciniphila quantification

The abundance of *A. muciniphila* in fecal samples and human tissues was extracted as genomic DNA (gDNA), cDNA Reverse Transcription and quantified by quantitative real-time PCR (qRT-PCR), in accordance with the method reported in a previous study 12 (**Supplementary Table 1**). Human fecal samples were collected from polyps (n = 46), adenomas (n = 38) and CRC (n = 42) patients (cohort 2) corresponding to the tissue samples from the same patients. The characteristics of the patients are listed in **Supplementary Table 2**. All the patients including those with polyps, A-CRA and CRC were first diagnosed and then histologically confirmed. Exclusion criteria were predefined as follows: diabetes, hypertension, coronary heart disease, and history of CRC.

### Azoxymethane (AOM)-initiated and dextran sulfate sodium (DSS)-promoted CRC mouse model

The mouse model AOM/DSS-induced CRC was established as previously described 26. Briefly, on day 1, the mice were injected with AOM (12.5 mg/kg, i.p.). After 1 week, the mice were given drinking water containing 2.5% DSS (International Lab, Chicago, IL, USA) for 7 days, followed by tap water for 14 days for recovery. This cycle was repeated twice.

### Pseudo-Germ-free mouse model

The mice were treated for 4 weeks of antibiotic solution (Abx), which contained ampicillin (1 mg/ml), neomycin (1 mg/mL), metronidazole (1 mg/ml), and vancomycin (0.5 mg/mL), all dissolved in the sterile drinking water as previously described 11. After four weeks, Abx treatment was stopped, and as previously described, the mice were treated with AOM and DSS combined with *A. muciniphila* (1×10^8^ colony forming units) or vehicle (E. coli MG1655 or the same volume of phosphate buffer saline) every day till neoplastic lesions developed. *A. muciniphila* was grown under anaerobic conditions at 37ºC overnight prior to further administration as described 8. Illness signs were monitored daily and body weight was recorded weekly.

### Mouse strains and breeding

*Apc^Min/+^* C57BL/6J mice were obtained from the Jackson Laboratory and crossed into *Apc^Min/+^* mice and wild-type littermates. All animals were kept under specific pathogen-free conditions in filter-top cages. Genotyping was performed at 4 weeks by PCR for AhR^fl/fl^ mice and villin-CreER mice, as described previously 27,28. We subsequently crossed mice with a floxed AhR^-/-^Cre locus to *Apc^Min/+^* mice to restrict AhR deficiency to *Apc^Min/+^* mice.

In total, all the mice were obtained from Shanghai Super-B&K Animal Laboratory Co., Ltd. (Shanghai, China) with Certification No. SCXK 2016-0016. All animals were housed under specific pathogen-free conditions in accordance with the Chinese Experimental Animals Administration Legislation.

### Histology and immunohistochemistry

The whole intestine was removed immediately from the sacrificed mouse, washed with ice-cold PBS, and opened longitudinally as previously described 11. The number, location, and size of visible adenomas throughout the intestine were measured. Tumors were grouped based on sizes < 2 mm, 2-4mm and > 4 mm. Tissue sections were fixed in 10 % formalin, paraffin-embedded, and stained with hematoxylin and eosin (H&E) for pathological evaluation by a pathologist blinded to the experimental groups. Histopathology of neoplastic lesions and degree of dysplasia were assessed according to the criteria and classification system of colonic adenomas.

### Immunohistochemistry (IHC) and TUNEL assay

Mouse tissues were harvested, fixed in 10% formalin overnight, and then processed for IHC. Human tissues were obtained and prepared as previously described 29. H&E and IHC staining were preformed using standard procedures. TUNEL assay was performed as previously described 30, following the manufacturer's instructions (BD Biosciences).

### RNA-seq and analysis

Total RNA was isolated from colon tissue with trizol. The RNA-seq library was prepared by Beijing Genomic Institution (BGI). Sequence reads were obtained using BGIseq500 and successfully mapped to mouse genome. For gene expression analysis, the matched reads were calculated and then normalized to fragments per kilobase million. Fold changes were calculated for all possible comparisons, and a 1.2-fold cutoff was used to select genes with expression changes. KEGG pathway analysis was performed using the R package, using significantly differentially expressed genes (*P* < 0.05) as target genes. The data mining and figure presentation process, including KEGG, the heatmap, and clustering, are all done by the BGI in-house customized data mining system called Dr.Tom (http://report.bgi.com).

### CRISPR/Cas9-mediated knockout of AhR

The genomic sequence of AhR was loaded at ensemble.org. mit.edu to design CRISPR. sgRNA strands for nuckase as previously described 31,32. Briefly, oligonucleotides targeting AhR (only one exon) were subcloned into LentiCRISPR v2. Lentivirus was produced by transfecting these constructs with psPAX2 and pMD2.G (Addgene) into HEK293T cells; meanwhile, DLD-1 and NCM460 cells were infected. Puromycin was used to weed out CRISPR-negative cells. The isogenic single-cell clones were obtained in a 96-well plate using serial dilution method. Single clones of transduced cells were screened for indels in AhR coding sequence by locus PCR/Sanger sequencing, and RT-PCR. Those without full-length AhR expression were used to perform further experiments.

### Organoid culture

Using small intestinal epithelial cells, the organoid culture was performed as described previously 33. Briefly, the organoids were cultured in matrigel with advanced DMEM/F12 medium (Invitrogen) supplemented with 50 ng/mL EGF (Invitrogen), R-Spondin1 conditioned medium (R&D Systems), and 100 ng/mL Noggin (Peprotech). The culture was passaged once, and the crypt length was measured under a dissecting microscope. Organoid cell proliferation was detected using the Click-iT EdU Imaging System (Invitrogen).

### Formation of colonospheres

The ability of cell lines to form spheres in the suspension was evaluated as described 34. Briefly, primary colonospheres were generated by incubating the limited number of NCM460 and DLD-1 cells, at a concentration of 100 cells per 200 μL in serum-free stem cell medium (SCM) containing DMEM/F12 (1:1) supplemented with B27 (Life Technologies, Gaithersburg, MD), 20 ng/ml EGF (Sigma, St Louis, MO), 10 ng/ml fibroblast growth factor (Sigma), and antibiotic-anti-mycotic in 24-well ultra-low-attachment plates (Corning Inc, Lowell, MA) for 10 days. The colonospheres formed at the end of the incubation were centrifuged (1000 rpm), dissociated with 0.05% trypsin/EDTA using a 22 gauge needle, and then filtered through a 40 μM sieve to obtain single cell suspension. The number and size of colonospheres formed after 5 days were evaluated by a light microscope.

### Western blot analysis

Whole cell lysates for Western blot analysis of AhR, β-catenin, TCF4, LEF1, and β-actin expression were prepared as previously reported 35. Briefly, the cells were maintained on the ice in lysis buffer for 2 hours before being collected with a cell scraper. The sample was centrifuged, and the supernatant was collected and stored at -80°C. Densitometric analysis was performed using the Scion Imaging application (Scion Corporation), with β-actin as the internal reference.

### LC-MS/MS quantification of Trp metabolites

Tumor homogenates were harvested after mixing tumor tissue pieces (50-300 mg) with Ultra-Turrax (IKA-T10, Sigma Aldrich) in PBS. Trp and Kyn concentrations were quantified by LC/MS-MS or HPLC-UV analysis after treatment with acetonitrile or trichloroacetic acid as described previously 36.

### Statistical Analysis

All the numerical data were presented as means ± standard deviation. All the statistical analyses were performed using GraphPad Prism 6 (GraphPad Software) and SPSS 23.0 (Chicago, IL, USA). A two-sided unpaired Student's t-test with Benjamini-Hochberg correction was used to compare the two groups. The differences among the several groups were analyzed using one-way analysis of variance (ANOVA) followed by Duncan's test. They were used for the analyses of colon tumor numbers, IHC, Western blot, Elisa, cell viability, quantitative RT-PCR, and transwell migration. The Wilcoxon rank-sum test was used for gut microbiome analysis of mice. Data are presented as three independent experiments at least. Significant probability values were indicated as *P < 0.05, **P < 0.01; ^#^*P* < 0.05, ^##^*P* < 0.01*.*

### Data availability

The sequenced data reported in this article have been submitted to the NCBI Sequence Read Archive (SRA) under accession no. PRJNA954981.

## Results

### 1. A. muciniphila abundance is negatively correlated with the development of colon cancer in both humans and mice

**Figure 1:**
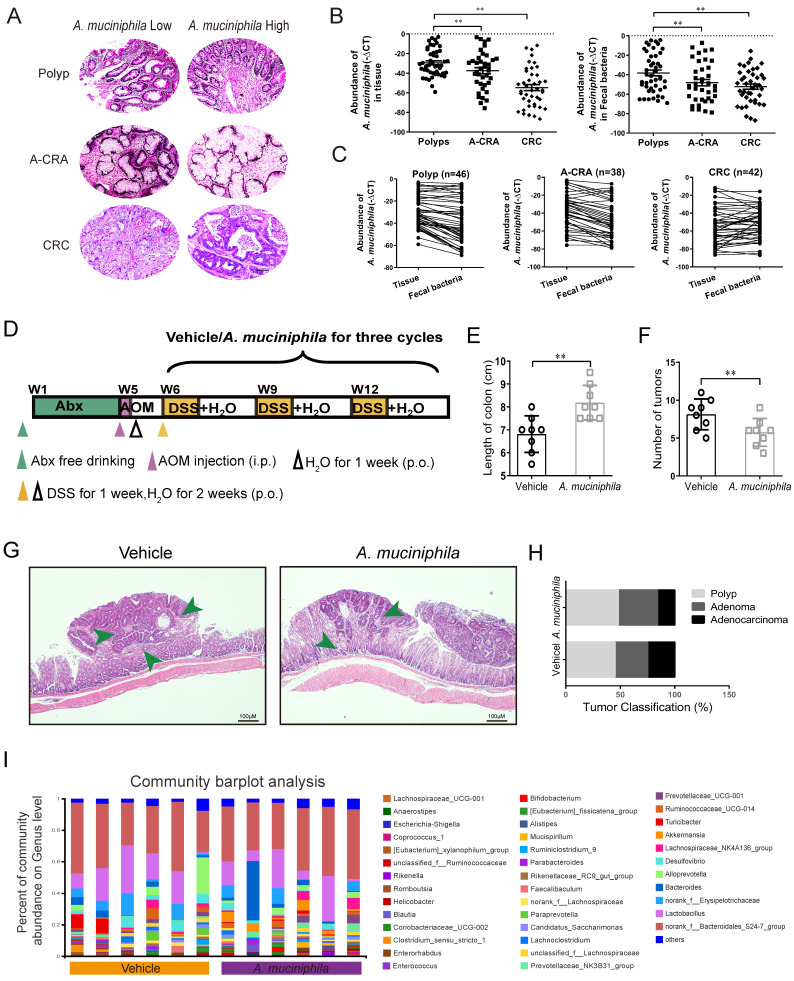
***A. muciniphila* abundance decreases in human CRC tissues and up-regulating *A. muciniphila* inhibits CRC *in vivo*.** (**A**) Polyp, A-CRA, and CRC tissues with decreased abundances of *A. muciniphila*. Scale bar, 100 mm. (**B**) Expression levels of *A. muciniphila* in Polyp, A-CRA, and CRC tissues and their fecal bacteria were analyzed by qRT-PCR. Sample numbers for the three groups were described as Materials and methods. **P < 0.01, Kruskal-Wallis test (**C**) Expression of *A. muciniphila*. in matched Polyp, A-CRA, and CRC tissues and their corresponding fecal bacteria. The bold horizontal bar represents mean expression levels (**D**) Experimental design of the intragastric administration and grouping. Mice (aged 4 weeks) were treated with Abx from W1 to W4, then injected with AOM (12.5 mg/kg, i.p.) at the first day of W5, and provided drinking water for 1 week, and three cycles with DSS and drinking water for 3 weeks as described in the Methods section. During the treatment, *A. muciniphila* and Vehicle (E. coli MG1655 or the same volume of phosphate buffer saline) were orally administrated (10^8^ CFU/mice). (**E**, **F**) Effects of *A. muciniphila* on colon length (**E**) and number of tumors (**F**) in pseudo-GF/AOM/DSS mice. Data of eight mice per experimental group are expressed as means ± SD, with Welch's correction through one-tailed t-test. **P < 0.01 (**G**) Typical adenomatous intestinal polyps with the early invasion of neoplastic glands into the muscular layers were observed in *A. muciniphila* infected pseudo-GF/AOM/DSS mice. This typical regressive change is observed throughout the intestine in the mice. Green arrows indicate adenocarcinoma cells. Scale bars, 100 μM. (**H**) Histology evaluation of tissues of polyps, adenoma, and adenocarcinoma. (**I**) A stacked bar plot of gena of 16S rRNA sequencing for fecal samples collected from model animals (Vehicle; n=6), *A. muciniphila* infected mice (*A. muciniphila*; n=6).

*A. muciniphila* has been verified as a promising probiotic in metabolic disorders, such as diabetes and obesity 37-39. Herein, we quantified the abundances of *A. muciniphila* in the tumor tissues from 126 patients with intestinal polyps, adenomas (A-CRA), and adenocarcinomas (CRC) (**Figure 1A and Supplementary Table 2, 3**). gDNA analysis showed that the abundance of *A. muciniphila* decreased both in tumor tissue and in faecal bacteria with cancer progression (**Figure 1B**). In addition, we showed that *A. muciniphila* abundance was decreased in tumor tissue compared to their corresponding adjacent normal mucosa, suggesting that *A. muciniphila* significantly decreased with the progress of tumourigenesis (**Figure 1C**). Taken together, these findings suggested *A. muciniphila* palyed an important role during the “adenoma-carcinoma” sequence in CRC.

Gut microbial imbalance can directly contribute to tumorigenesis in both *Apc*-mutant multiple intestinal neoplasia (Min) mouse model and AOM/DSS CRC mouse model 26. To investigate whether the tumorigenesis in CRC mice was inhibited by *A. muciniphila*, we used a pseudo-GF mediated AOM/DSS mouse model** (Figure 1D)**. We observed that *A. muciniphila* significantly decreased the number and volume of colonic tumor in the AOM/DSS mouse model, as compared with the Vehicle group **(Figure 1E-F)**. Moreover, histological analysis revealed much lower tumor grades in *A. muciniphila*-treated mice, compared to those in the Vehicle group **(Figure 1G-H)**. Notably, compared with the Vehicle group, a higher abundance of *A. muciniphila* inhibited the progression of CRC including in EM imaging data (**Supplementary Figure 1A**). We further used 16S rRNA sequencing and analysis of electron microscopy to detect the protective effect of *A. muciniphila* on the colon in the above model (**Figure 1I and Supplementary Figure 1B-E**).

### 2. A. muciniphila-attenuated tumorigenesis is associated with tryptophan-mediated AhR pathway

**Figure 2:**
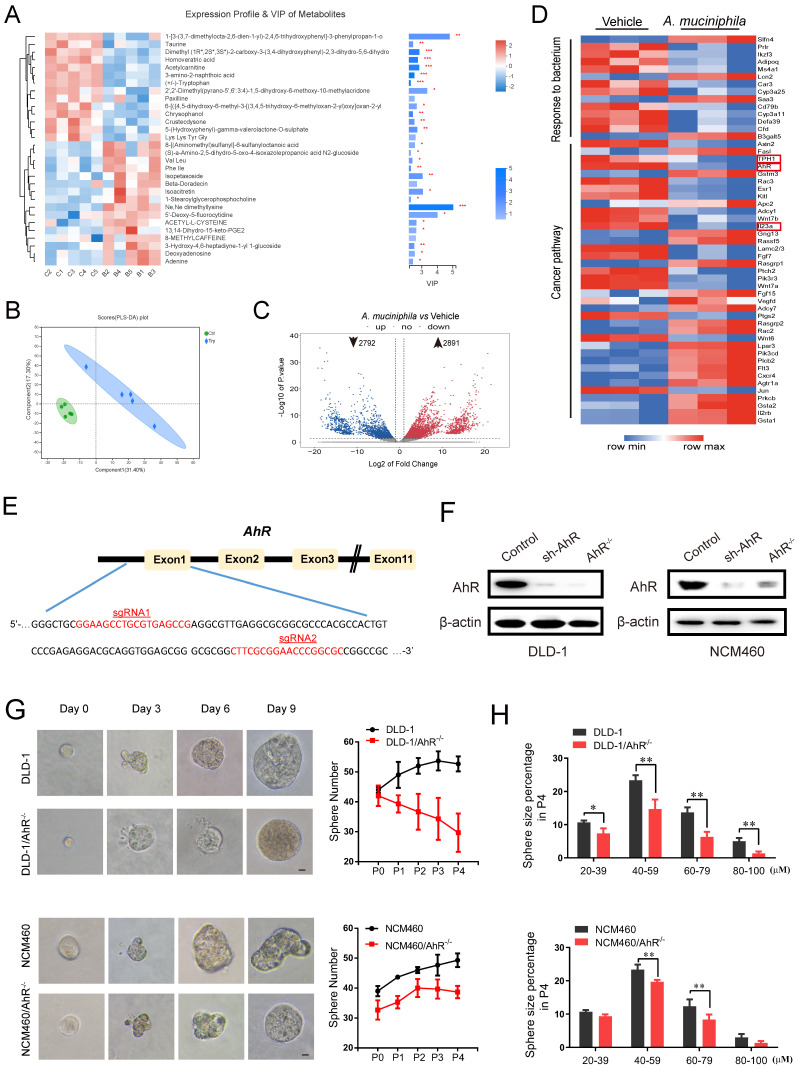
***A. muciniphila* inhibits CRC development via AhR.** (**A**) Top 30 metabolite analysis of the gut microbiome of mice in the combination *A. muciniphila* group vs. the Vehicle group by the Wilcoxon rank-sum test. Data are expressed as mean ± SD. * 0.01 < *P* ≤ 0.05, ** 0.001 < *P* ≤ 0.01, *** *P* ≤ 0.001, Two-sided Hypotheses. (**B**) GO terms and KEGG pathways. PLS-DA plots of metabolite based on the Bray-Curtis distance. (**C**) RNA sequencing analysis of DEGs in the tumor tissues derived from the mice model described in Figure 1D (n = 3). Volcano plot: p-values are plotted against the log2 of the change in corresponding RNA expression level of AhR in *A. muciniphila*-infected pseudo-GF/AOM/DSS group versus that in pseudo-GF/AOM/DSS group. Dashed vertical lines indicate cut-offs for differential expression. Dashed horizontal lines indicate the cut-off for adjusted p-value <0.05 determined with DESeq2. (**D**) Unsupervised cluster analysis. (**E**) Two CRISPR nuclease sgRNA designed for AhR. A schematic illustrates the first three and the last intron-exon organization of the AhR gene. AhR KO cells were produced by CRISPR/Cas9-induced mutations resulting in a frameshift and premature stop codon. The locations of sgRNA target sequences and the alignment of amino acid sequences, showing changes introducing a premature stop codon, are noted. (**F**) Western blot analysis of AhR expression. In DLD-1 and NCM460 cells, proteins were collected from AhR knockout cells (AhR*^ -/-^*), AhR sh-RNA cells and Control cells (CN). (**G**) DLD-1/AhR*^ -/-^* cells and NCM460/AhR*^ -/-^* cells were cultured *in vitro* through sphere-formation assay. Left panels are representative phase contrast photos (scale bar, 50mm). Data in right panels represent the average of sphere numbers from three independent experiments. (**H**) The number and size of one-generation spheres were quantified at 3 days after plating by counting viable spheres per well. Data from triplicate experiments are presented as mean ± SD. *P < 0.05, **P < 0.01, t test*.*

There are numbers of agreement on the association with specific gut bacteria mediated metabolites and alteration of cellular signal and transferring proteins during tumorigenesis 11, 40. As shown in **Figure 2A**, it was found that 30 metabolites were highly predictive for the differentiation between the two groups. Among the 30 key metabolites, Trp were significantly decreased in *A. muciniphila* group compared with Vehicle group. Moreover, the principal-coordinate analysis (PLS-DA) based on Bray-Curtis distances indicated that the *A. muciniphila* group was separated from the Vehicle group (**Figure 2B**), which indicated that *A. muciniphila* caused a change in the community composition of gut metabolites.

As shown in** Figure 2C**, *A. muciniphila* down-regulated 2792 and up-regulated 2891 genes in pseudo-GF/AOM/DSS mice. Unsupervised cluster analysis further revealed that the down-regulated genes were enriched in cancer pathways, and the up-regulated genes in gut barrier function (**Figure 2D**). Of note, TPH1, AhR and IL23, which are associated with Trp-medicated AhR signaling pathway, showed the most prominent changes in expression after *A. muciniphila* infection (**Figure 2D**).

Previous studies have reported that AhR deletion enhanced the proliferation of intestinal epithelia cells (IECs) and disrupted intestinal epithelial barrier function 41. We evaluated the expression level of AhR in several CRC cell lines (**Supplementary Figure 2A**). AhR levels in DLD-1 cells and NCM460 cells were the highest (**Supplementary Figure 2A**). Then, we knocked out AhR gene in the cells using CRISPR/Cas9 technology. Using the online guiding RNA design software (https://zlab.bio/guide-design-resources), two optimal CRISPR nuclease sgRNAs targeting the exon region of AhR genome were designed, subcloned into the LentiCRISPRv2 plasmid (**Figure 2E**), and were validated effectively in HEK293T cells (**Supplementary Figure 2B**). After a series of screening experiments (**Supplementary Figure 2C**), Western blotting analysis further confirmed the specific deletion executed by CRISPR/Cas9 sgRNA. We named the cells with AhR deletion as DLD-1/AhR^-/-^ cells and NCM460/AhR^-/-^ cells (**Figure 2F and Supplementary Figure 2D**).

The cancer stem cell (CSC) theory postulates that a small subset of tumor cells is responsible for tumor initiation and progression 42. These cells, also referred as tumor-initiating cells, can self-renew and generate all the cells comprising the tumors 43. To explore the proliferative capacity of AhR to colorectal cells and normal colon epithelial cells, regeneration or secondary colonospheres formation assay was carried out (**Figure 2G-H**). The numbers (**Figure 2G**) and sizes of colonospheres (**Figure 2H**), and CSC marker (**Supplementary Figure 2E**) were significantly decreased in both DLD-1/AhR^-/-^ cells and NCM460/AhR^-/-^ cells, compared with those in DLD-1 cells and NCM460 cells. In particular, compared with NCM460 cells, AhR deletion led to death of DLD-1 colonospheres with the increase of passage times likely due to the cytostatic effect on cancer stem cell (not IECs). These results highlighted the potential of AhR in blocking the CRC cell growth in both concentration- and time-dependent manners. Next, we chose DLD-1/AhR^-/-^ cells as the cell model for subsequent animal experiments.

### 3. AhR regulates the development of CRC

**Figure 3:**
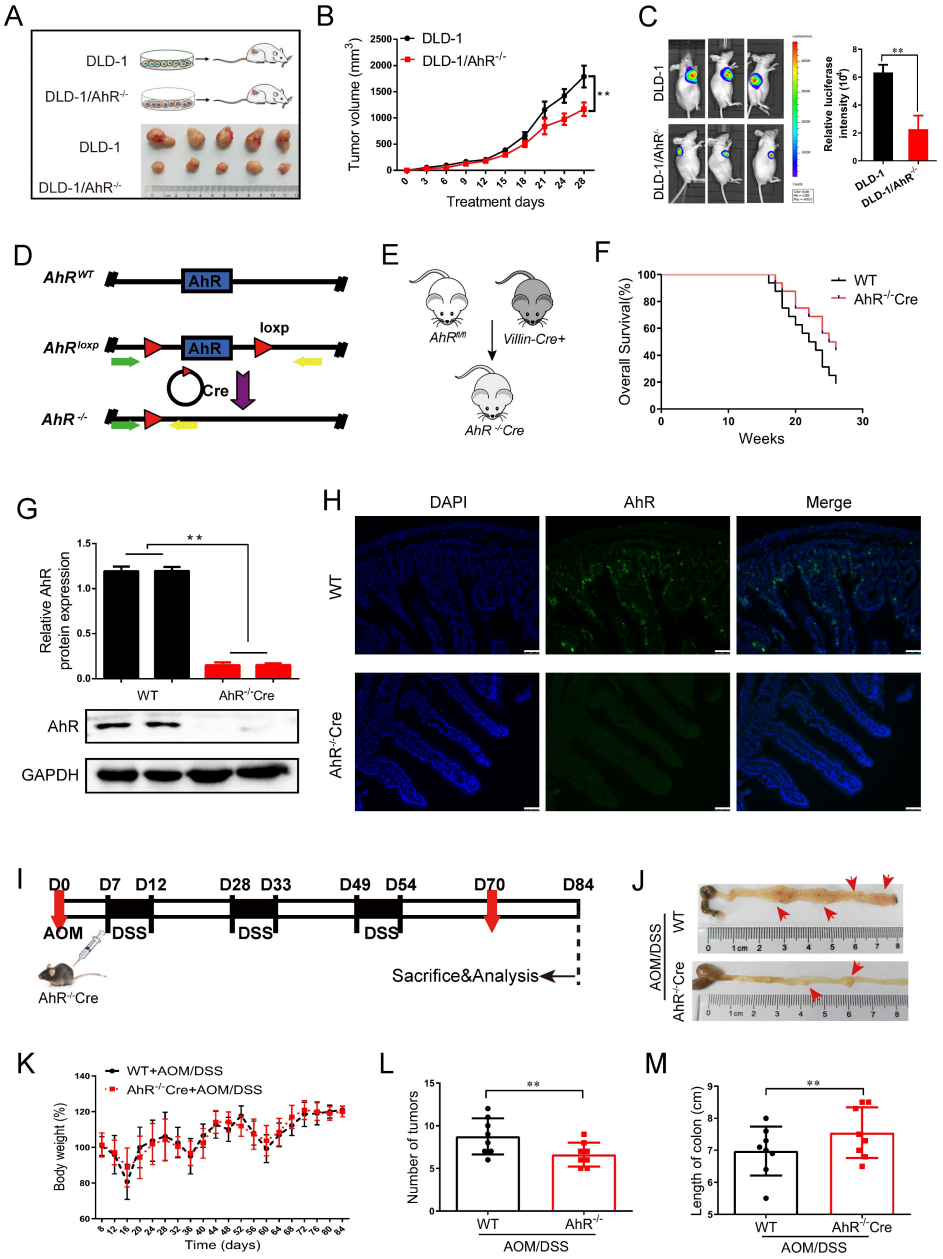
** AhR deficiency inhibits the development of CRC.** (**A**) Schematic diagram showing the experimental design in the mouse xenograft model. (**B**) The function of AhR deficiency on the growth of DLD-1 cell xenografts in the nude mice was examined. (**C**) Luciferase imaging of the mice with DLD-1 or DLD-1/AhR*^ -/-^* xenografts at 28 days after tumor cell implantation. The quantification of luciferase intensities in tumors in two groups. (**D**) Generation of AhR^ fl/fl^ IECs and mice. Restriction sites and expected restriction fragment lengths are indicated. (**E**) Breeding strategy to determine tumor-initiating potential of AhR^+^ cells. (**F**) Survival analysis of AhR*^ -/-^* Cre mice and WT mice (n=16/group). (**G**) Western blotting for AhR protein with IEC lysates isolated from fl/fl and WT mice. Data from triplicate experiments are presented as mean ± SD. **P < 0.01. (**H**) Immunofluorescence micrographs of AhR (green) and DAPI (blue) expression in the colons of WT and AhR*^ -/-^* Cre mice. Scale bars, 100 μM. (**I**) Experimental protocol used for the induction of the CAC in the AhR*^ -/-^* Cre mouse model. (**J-M**) Effects of AhR deficiency on CRC development evaluated with microscopic images (**J**), body weight (**K**), number of intestinal polyp colon length (**L**), and colon length (**M**) in WT and AhR*^ -/-^* Cre mice. Data of eight mice per experimental group are shown as means ± SD, with Welch's correction through one-tailed t-test. **P < 0.01.

To investigate the CRC-suppressing effect of AhR *in vivo*, we subcutaneously transplanted DLD-1/AhR^-/-^ cells into Balb/c nude mice, finding that knockdown AhR suppressed tumor growth (**Figure 3A**). As expected, tumor growth slows and tumor cell apoptosis increases significantly in DLD-1/AhR^-/-^ tumour-bearing mice compared with that in DLD-1 tumour-bearing mice (**Figure 3B-C; Supplementary Figure 3; and Supplementary Table 4**).

To explore the effects of AhR deficiency on the progression of CRC, the AhR^fl/fl^ Villin^cre^ mice were generated and validated by quantitative polymerase chain reaction (qPCR) and Western blotting. We generated a conditional knockout (KO) allele, AhR^fl^, using conventional gene targeting and crossed homozygous AhR^ fl/fl^ mice with recombination of the AhR ^flox^ allele in the IECs in VillinCre mice expressing Cre recombinase (Cre+) (**Figure 3D**). We then constructed AhR^-/-^Cre mice and wild-type controls (**Figure 3E**). Given that AhR occupies the loci associated with endoderm development and epithelial differentiation, we next determined whether AhR is required during the intestinal epithelium growth in the mice. It is noteworthy that, we observed no obvious morphological changes in the intestines and survival rate, even at weeks or months after AhR deletion (**Supplementary Figure 4A and Figure 3F**). The IECs in AhR^-/-^Cre mice displayed near-complete loss of AhR by Western blotting (**Figure 3G**) and IHC (**Figure 3H**), indicating a high KO efficiency.

We sought to determine the effect of AhR deficiency on adenocarcinoma formation in the intestines of AOM/DSS mice. AhR deficiency induced oncogenic potential combined with repeated cycles of DSS treatment. The establishment of the AOM/DSS mouse model is shown in **Figure 3I**. Interestingly, the proliferation of adenocarcinoma cells of AOM/DSS mice was inhibited by AhR deficiency (**Figure 3J**). After AOM/DSS feeding, body weight loss (**Figure 3K**), low fodder consumption (**Supplementary Figure 4B**), and bloody stools were observed (**Supplementary Figure 4C**). As expected, compared to that in the AOM/DSS- control group, colon adenoma size was significantly reduced by 24.28% in AOM/DSS-AhR^ -/-^Cre group (**Figure 3L-M and Supplementary Table 5**). The increase of Immunohistochemical (IHC) staining in TUNEL, BCL-2 and Caspase-3 reflected AhR deficiency downregulation induced apoptosis in the proliferation of adenocarcinoma cells of AOM/DSS mice (**Supplementary Figure 4D).** These results suggested that AhR deficiency protected against AOM/DSS-induced colitis-associated CRC.

### 4. AhR deficiency inhibits CRC development in the Min mouse model

**Figure 4:**
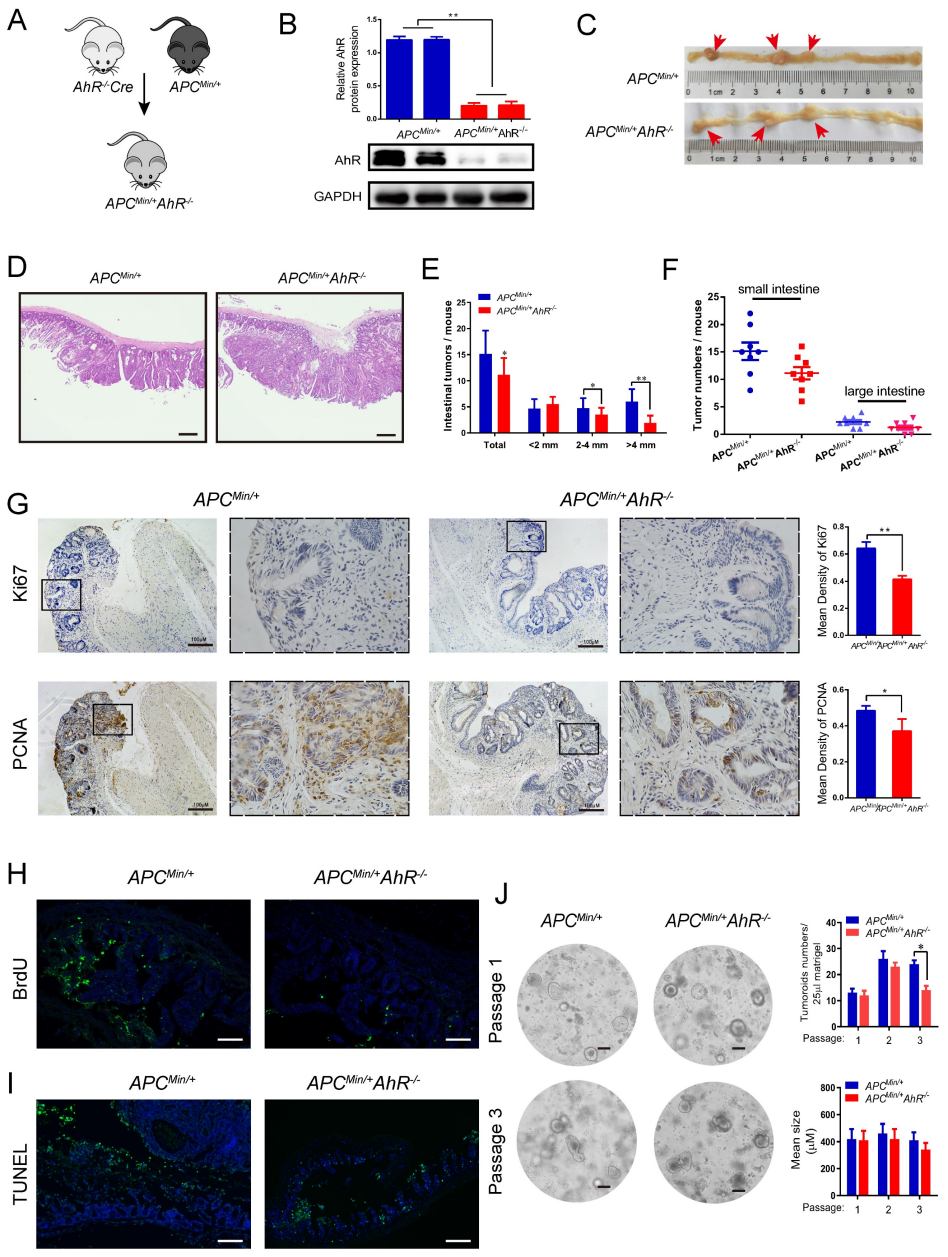
** AhR deficiency inhibits CRC development in the multiple intestinal neoplasia (Min) mouse model.** (**A**) Breeding strategy to generate* Apc^Min/+^* AhR*^ -/-^* and *Apc^Min/+^* mice. (**B**) Western blotting for AhR protein with IEC lysates isolated from *Apc^Min/+^* AhR*^ -/-^* and *Apc^Min/+^* mice. (**C-F**) Microscopic effects of AhR deficiency on CRC. (**C**) Representative H&E staining images of colon tissue. (n=8/group). Scale bar: 100 μM. (**D**) Tumor size distribution in *Apc^Min/+^* AhR*^ -/-^* and *Apc^Min/+^* mice. (**E**) Tumor size distribution in the intestine listed and compared with *Apc^Min/+^* mice. (**F**) Numbers of tumors in the small intestine and colon from different groups. (**G-I**) Immunohistochemical staining with an antibody against PCNA, Ki67, BrdU and TUNEL in *Apc^Min/+^* AhR*^ -/-^* and *Apc^Min/+^* mice. Scale bar: 100 μM. (**J**) Representative pictures of tumor organoids originating from small IECs in *Apc^Min/+^* AhR*^ -/-^* and *Apc^Min/+^*mice. After isolation, organoids were kept in ENR medium (containing EGF, Noggin and RSPO1). At 7 days after isolation, the organoids were passaged and EN medium (containing EGF and Noggin) was used for organoids culture. Pictures were taken at 7 days after passaging in case of passage 1 and passage 3. Scale bar: 100 μM. Data of eight mice per experimental group are expressed as means ± SD, with Welch's correction through one-tailed t-test. *P < 0.05, **P < 0.01.

We explored the effect of AhR in the mouse model of adenomatous polyposis coli* (Apc)*/Wnt-driven CRC. Similar as before, we crossed AhR CreER mice with *Apc^Min/+^* mice to generate *Apc^Min/+^* AhR*^ -/-^* mice and treated them with tamoxifen (**Figure 4A**). The expression of AhR after tamoxifen treatment was demonstrated by Western blotting using lysates of IEC samples (**Figure 4B**). Compound mutant (*Apc^Min/+^* AhR*^ -/-^*) mice showed significant decrease in tumor multiplicity when compared with the single transgenic littermates (**Figure 4C**). Surprisingly, the *Apc^Min/+^* AhR*^ -/-^* mice showed a low incidence of intestinal adenoma than *Apc^Min/+^* controls (**Figure 4C**), which is consistent with the result in a previous study 11. We also evaluated the dysplasia in the colonic crypts using H&E staining (**Figure 4D**). Similarly, we found that the *Apc^Min/+^* AhR*^ -/-^* mice showed a lower incidence of adenoma in both the small and large intestines (**Figure 4E-F**) when compared with the *Apc^Min/+^* controls. We then examined the location and expression of Ki67 and PCNA by immunohistochemistry in the tumor tissues of *Apc^Min/+^* mice and *Apc^Min/+^* AhR*^ -/-^* mice. Compared with that in the control group, the expression of Ki67 and PCNA in the nuclei of IECs decreased in the *Apc^Min/+^* AhR*^ -/-^* mice (**Figure 4G**). In addition, cell proliferation was significantly decreased in the *Apc^Min/+^* AhR*^ -/-^* mice, which were detected by BrdU reactivity (**Figure 4H**). More prominent apoptosis was found in tumor cells of IECs the *Apc^Min/+^* AhR*^ -/-^* mice than in the WT mice by TUNEL staining (**Figure 4I**).

AhR helps maintain a stem cell-like expression signature in tumor cells 44. So, we generated tumor organoids using single cells derived from the adenomas of *Apc^Min/+^* and *Apc^Min/+^* AhR*^ -/-^* mice. Organoid culture of the wild-type (APC) mouse-derived IECs showed buds from cysts formed mini-crypt structures (**Figure 4J** left), which is as originally reported 41. We further examined the IEC proliferation by the number of organoids (**Figure 4J** up), and the IEC activity by the mean size of organoids (**Figure 4J** bottom). Taken together, AhR could initiate, but not maintain the development of tumoroid lesions. These results were in line with those observed in the *Apc^Min/+^* mice that AhR deficiency decreased the number and size of adenoma, but showed no effect on its persistence afterward.

### 5. AhR activates the Wnt/β-catenin signaling in CRC cells

**Figure 5:**
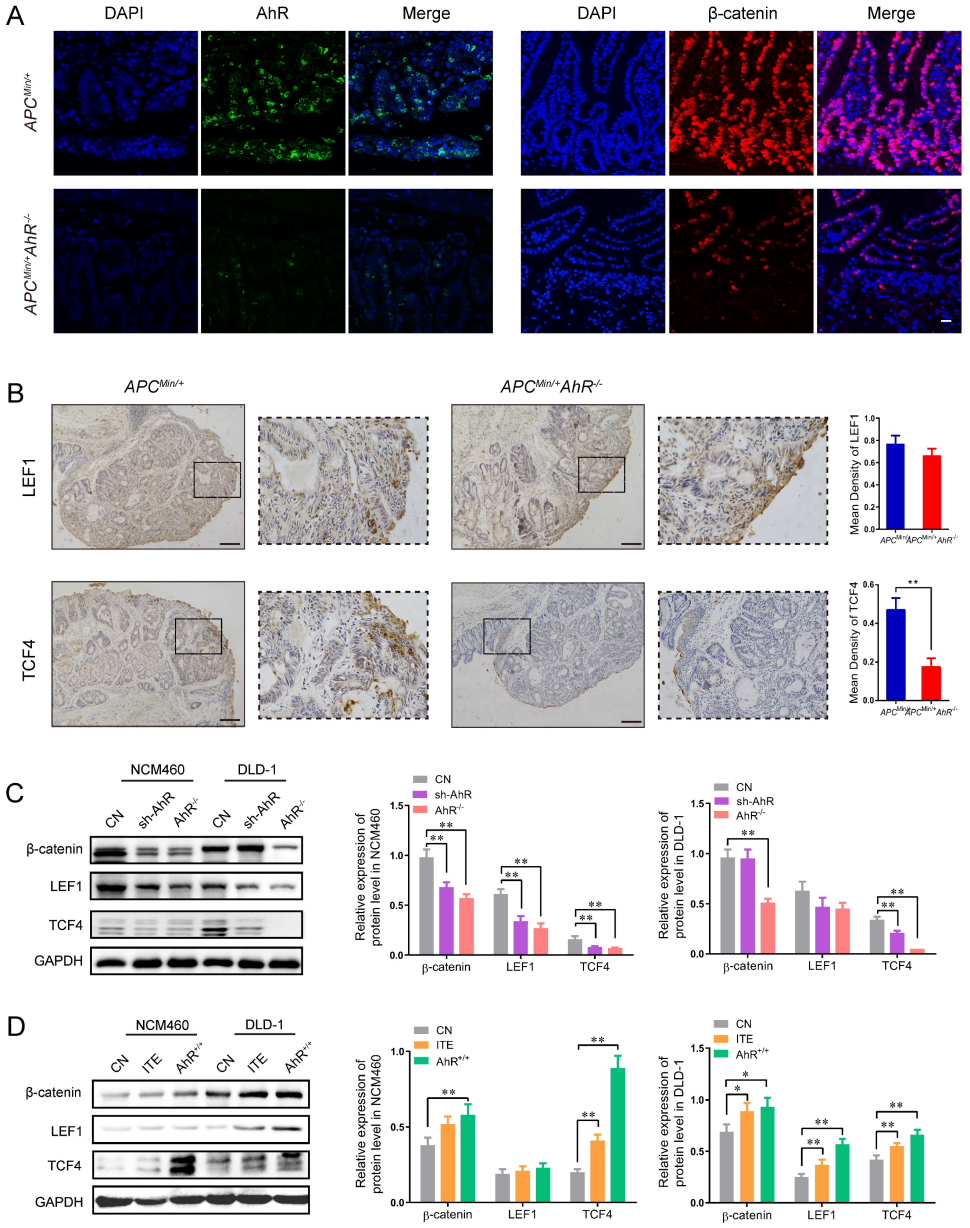
** AhR activates the Wnt/β-catenin signaling pathway in CRC Cells.** (**A**) Immunofluorescence micrographs of AhR (green) and β-catenin (red) expression in the colon tissues of *Apc^Min/+^* AhR*^ -/-^* and *Apc^Min/+^* mice. Scale bar: 100 μM. (**B**) Immunohistochemical staining with an antibody against c-Myc and cyclinD1 in *Apc^Min/+^* AhR*^ -/-^* and *Apc^Min/+^* mice. Scale bar: 100μM. (C) Western blotting showing expression levels of β-catenin, LEF1, and TCF4 in AhR KO, AhR sh-RNA, and WT in DLD-1 and NCM460 cells as described in Materials. (**D**) Western blotting showing expression levels of β-catenin, LEF1, and TCF4 in DLD-1 and NCM460 cells co-treated with ITE (10.0 μM, 24h), or AhR KO cells. Data from triplicate experiments are presented as mean ± SD, with Welch's correction through one-tailed t-test. *P < 0.05, **P < 0.01.

The above-mentioned findings raised the possibility that AhR may interfere directly with the expression of Wnt/β-catenin-targeted genes. Consistently, AhR and β-catenin staining was only observed in the tumor cells of *Apc^Min/+^* mice, but not in the lamina propria cells of *Apc^Min/+^* AhR*^ -/-^* mice (**Figure 5A**).

Since LEF and TCF are essential transcription factors in the Wnt/β-catenin pathway and mediators of tumor formation induced by *Apc* deficiency in the intestine 29, 35, we examined the localization and expression of LEF and TCF in the tumor tissues from the *Apc^Min/+^* mice and *Apc^Min/+^* AhR*^ -/-^* mice by IHC (**Figure 5B**). Compared with the *Apc^Min/+^* mice, the expression levels of LEF1 and TCF4 decreased in the nuclei of polyp IECs in the *Apc^Min/+^* AhR*^ -/-^* mice (**Figure 5B**). Compared with *A. muciniphila* higher tissues, tumor areas had more AhR^ +^ cells in *A. muciniphila* lower tissues, which positively correlated with the clinical stage of colorectal cancer (**Supplementary Figure 5A**). As expected, data from TCGA and TIMER (http://timer.cistrome.org/) analysis showed that AhR levels were positively related to the expression levels of human β-catenin, TCF1 and LEF4, some typical target genes of Wnt/β-catenin pathway (**Supplementary Figure 5B-C**).

To determine whether the antiangiogenic activity of AhR is linked to the Wnt/β-catenin signaling pathway, the expression levels of TCF1 and LEF4 were examined in DLD-1 and NCM460 cells. The expression levels of β-catenin, TCF1 and LEF4 in DLD-1 cells decreased significantly after AhR knockdown or knockout (**Figure 5C**). The Western blotting showed that AhR over-expression vector increased the expression levels of β-catenin, TCF1 and LEF4, which were also increased by ITE (an endogenous AhR ligand) **(Figure 5D)**. Taken together, these results suggested that AhR regulated TCF1/LEF4 genes involved in the Wnt/β-catenin signaling pathway in the colonic tumorigenesis models *in vivo* and *in vitro*.

### 6. Tryptophan availability modulates the growth of colorectal cancer in AhR‑-mediated Wnt/β-catenin pathway

**Figure 6:**
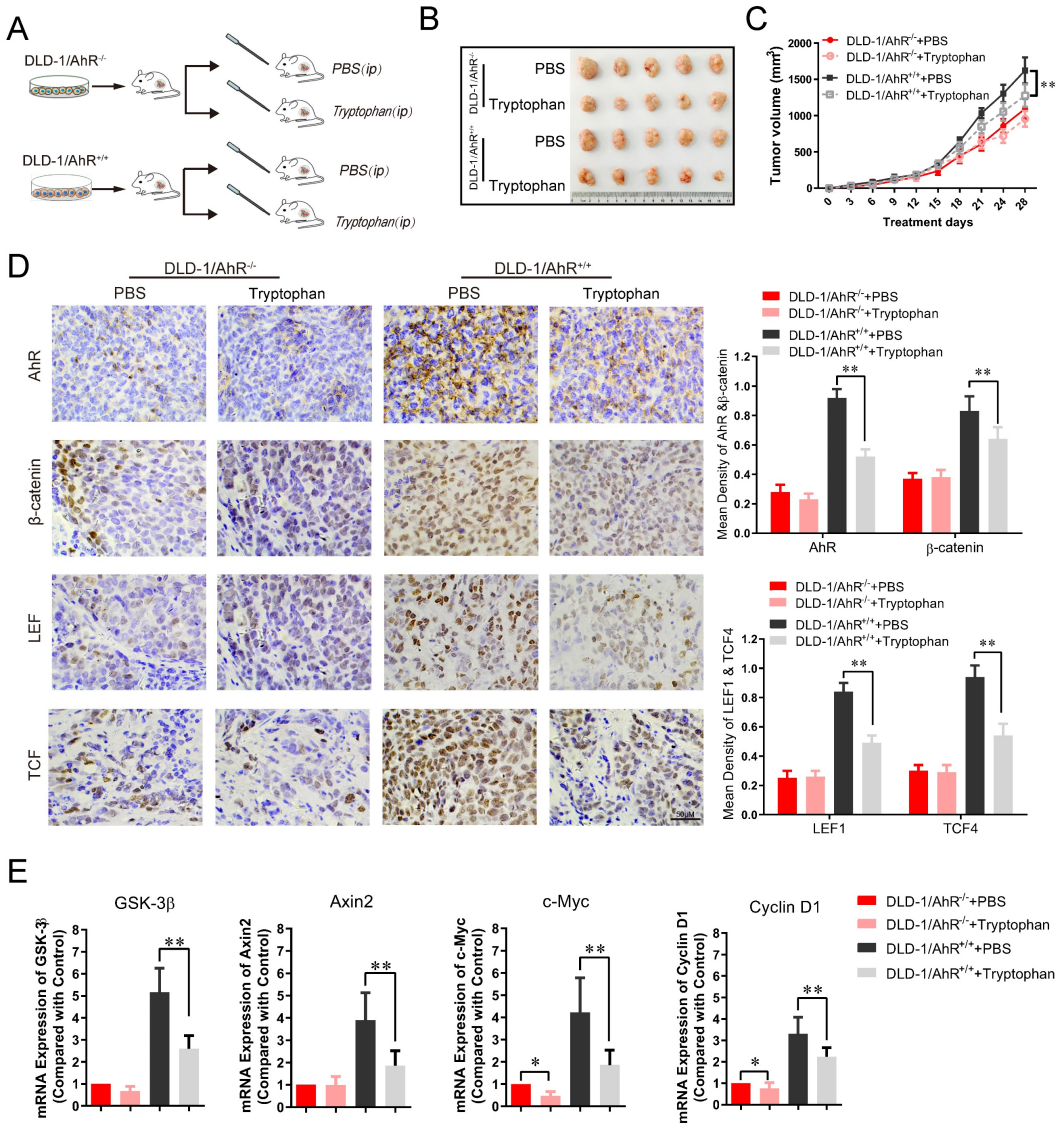
** Tryptophan inhibits subcutaneous xenograft of colorectal cancer through AhR mediated β-catenin/TCF/LEF pathway.** (**A**) Schematic diagram of the xenograft mouse model gavaged with Tryptophan (**B, C**) Representative data of tumors in the nude mice bearing DLD-1/AhR^-/-^ and DLD-1/AhR^+/+^ cells (1 × 10^6^ cells) treated with or without Tryptophan in different groups. N=5/group. Statistical analysis of tumor volume. (**D**) Immunohistochemical staining of AhR, β-catenin, LEF1, TCF4 in different groups. Scale bar: 100 μM. (**E**) The mRNA expression level of Wnt/β-catenin signaling downstream effector like GSK-3β, Axin2, c-Myc and cyclin D1 were evaluated in tumor tissues. Data are presented as the means ± SD of five animals per experimental group with Welch's correction, one-tailed t test. *P < 0.05, **P < 0.01.

To further assess the effects of Trp/AhR-mediated activation of Wnt/β-catenin pathway on colorectal tumor growth, DLD-1 cells were transfected with AhR over-expression plasmid and inoculated into the nude mice, followed by gavaged with Tryptophan (0.8%) for 4 weeks as previous described 45. Tumor growth *in vivo* was evaluated according to tumor volume and weight. The experimental design is shown in **Figure 6A**. In AhR deficiency CRC xenograft mouse model, the inhibitory effect of Tryptophan on tumorigenesis was not observed significant difference between the control and treatment groups (**Figure 6B-C**). No significant differences were found in tumor size (**Figure 6B**), tumor inhibition rate (**Figure 6C**), or AhR, β-catenin and TCF1/LEF4 expression (**Figure 6D**) between the DLD-1/AhR*^-/-^* control group and the DLD-1/AhR*^-/-^* + Tryptophan group. However, the above comparisons in all respects differed significantly in the common control (subcutaneous transplantation tumour of DLD-1 cells). Taken together, the tumorigenesis-inhibiting effect of Tryptophan depended, at least in part, on AhR activation. To further confirm that AhR plays a critical role in intestinal stem cells (ISCs) by calibrating their response to Wnt/β-catenin signals, more tests were carried out for their downstream effector of Wnt signalling pathway. qRT-PCR analysis revealed a markedly lower expression of total Axin2, GSK-3β and their downstream effector c-Myc and cyclin-D1 in Tryptophan intervention group, compared to that in the common control (**Figure 6E**). Downregulation of AhR also resulted in a marked reduction in Wnt/β-catenin signaling, which suggest that Trp/AhR-mediated Wnt/β-catenin pathway is intricately involved in the growth and maintenance of colorectal carcinoma.

### 7. Effect of A. muciniphila in the AhR-deficient mouse model

**Figure 7:**
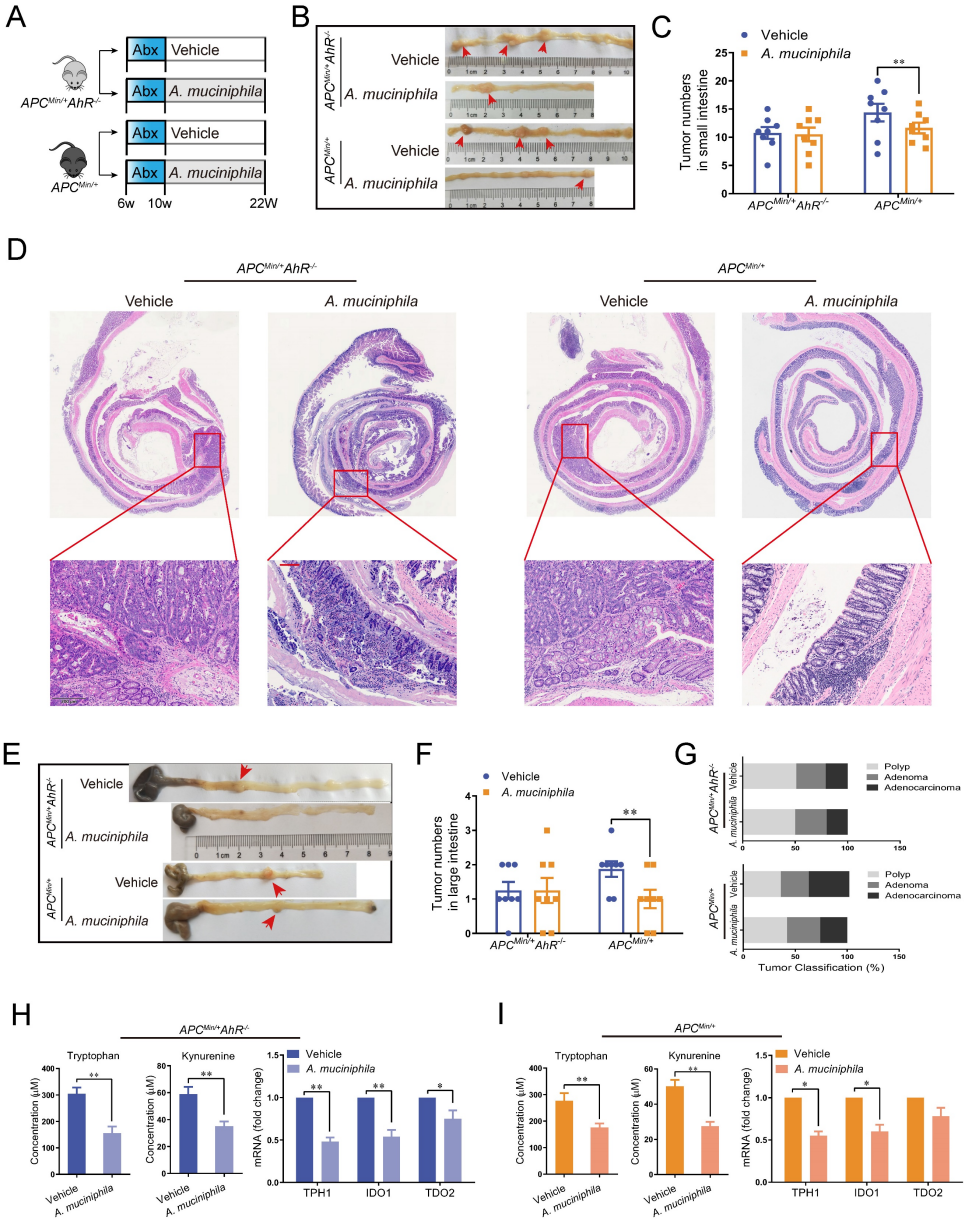
** Effect of *A. muciniphila* in AhR deficiency model.** (**A**) Schematic diagram showing the infection of *A. muciniphila* in *Apc^Min/+^* AhR*^ -/-^* or *Apc^Min/+^* mice. (**B**, **C**) Macroscopic images showing several polypoid and discoid colonic tumors in *Apc^Min/+^* AhR*^ -/-^* or *Apc^Min/+^* mice at 12 weeks after infection with *A. muciniphila*. Data from triplicate experiments are presented as mean ± SD. ANOVA test, **P < 0.01. (**D**, **E**) Number of intestinal polyps in the small intestine or colon in different groups. (**F**) Histology evaluation of colon tumors shown as polyps, adenoma, and adenocarcinoma. Data from triplicate experiments are presented as mean ± SD. ANOVA test, **P < 0.01. (**G**) Intestinal morphological changes were observed in mice fed with *A. muciniphila*, including reduced villi length and deeper crypt invaginations and analysed by representative H&E colon stainings. (**H**) Left: Trp and Kyn levels in the tumor tissue of *Apc^Min/+^* AhR*^ -/-^* mice infected with or without *A. muciniphila*. Right: qRT-PCR for the expression of Trp transporters and enzymes in the tumor tissues of *Apc^Min/+^* AhR*^ -/-^* mice infected with or without *A. muciniphila*. (**I**) Left: Tumor tissue levels of Trp and Kyn in *Apc^Min/+^* mice infected with or without *A. muciniphila*. Right: qRT-PCR for the expression of Trp transporters and enzymes in the tumor tissues of *Apc^Min/+^* mice infected with or without *A. muciniphila*. Data from triplicate experiments are presented as mean ± SD, with Welch's correction through one-tailed t-test. *P < 0.05, **P < 0.01.

Similar to that in the AhR deficiency CRC xenograft mouse model, the inhibitory effect of *A. muciniphila* on tumorigenesis was not impaired in AhR-deficient *Apc^Min/+^* mouse model, as shown by reduced tumor number in large intestine and small intestine (**Figure 7 A-G**). Similarly, a significant tumorigenesis-inhibiting effect of *A. muciniphila* was observed after feeding with *A. muciniphila* in *Apc^Min/+^* mouse, but the difference was not significant in AhR-deficient *Apc^Min/+^* mouse model (**Figure 7 E**). In addition, the tumor load/tumor number in the intestine, which is the main readout of this model, was significantly lower in *Apc^Min/+^* mouse model compared with that in AhR-deficient *Apc^Min/+^* mouse model after *A. muciniphila* treatment (**Figure 7 F**). Furthermore, histological analysis of colon revealed that *A. muciniphila* did not increase the malignancy of adenocarcinoma in the AhR-deficient *Apc^Min/+^* mouse model, compared to that in the *Apc^Min/+^* mouse model (**Figure 7 G and Supplementary Table 6**). To confirm *A. muciniphila*-mediated tryptophan metabolic activity *in vivo*, we collected intestinal tissue and plasma and measured concentrations of Kyn and Trp. We observed a concomitant decrease of Trp and Kyn in mRNA level, confirming a sustained inhibition of Trp degradation by *A. muciniphila in vivo* (**Figure 7 H-G and Supplementary Figure 6**).

## Discussion

In this study, we found that *A. muciniphila* could block the activation of tryptophan -mediated AhR/Wnt/β-catenin signaling pathway to reduce the ability of cells to proliferate, thereby inhibiting microbiota-driven differentiation of IECs that facilitates colonic tumorigenesis.

Microbiota disruption contributes to CRC pathogenesis 46,47. Epidemiological studies have shown that the abundance of *A. muciniphila* decreases significantly in patients with CRC 38. *A. muciniphila* is enriched in the fecal samples of inflammation-associated CRC mice 48. However, the mechanisms of *A. muciniphila*'s in inducing CRC are unclear. A recent study has revealed that the colonization of *A. muciniphilahas* increases the incidence of tumors in *Apc^Min/+^* mice, especially the tumors originating from the mucus layers and goblet cells 49. Remarkably, a large body of evidence has demonstrated that *A.muciniphila* and a specific outer membrane protein (eg. Amuc_1100 and Amuc_2172) inhibits tumourigenesis through the expansion and activation of CTLs, or reprogrammed colorectal tumour microenvironment (TME) 8,50. In the present study, *A. muciniphila* inhibited the development of CRC in three mouse models of nude xenograft, AOM sporadic CRC and *Apc^Min/+^*. In line with these findings in the mice, *A. muciniphila* abundance was also negatively correlated with tumor development in humans.

Trp is mainly obtained from food and has a variety of physiological functions. *In vivo*, Trp is mainly metabolized in the small intestines and central nervous system 51. Recent studies have investigated the involvement of Trp metabolites in the pathogenesis of IBD and CRC 52. For example, intestinal inflammation is attenuated in the mice injected with three *Lactobacillus* strains capable of metabolizing tryptophan, which suggests that Trp uptake can positively affect the intestinal structure 53. It has shown that formation of pre-CRC lesions is highly correlated with Trp metabolism, energy metabolism, polyamine metabolism, and the composition of the intestinal flora. With the in-depth study of metabolites and TME particularly the immune microenvironment,Trp-Kyn metabolic pathway is accepted as a pathogenic driver from IECs to CRC progression through T-cell 54. In this study, our results demonstrated that feeding *A. muciniphila* decreased the uptake of Trp in AOM/DSS mice and decreased the expression of AhR.

AhR, as a Trp receptor, can maintain mucosal homeostasis. We subsequently explored the molecular mechanism by which *A. muciniphila* regulates the function of AhR. In the intestines, Trp is mainly metabolized through three cancer-related pathways: the kynurenine pathway (KP), the serotonin pathway, and the protein synthesis pathway. Kyn production is catalyzed by three enzymes: IDO1, IDO2, and TDO2. Trp is finally transformed into Kyn by arylformamidase (AFMID) 55. Herein, we deleted AhR using either CRISPR or Villin-Cre-mediated to test the effect of AhR on CRC genesis. Our data showed that the growth of AhR-deficient CRC cells and IECs was inhibited significantly, suggesting that AhR expression is indispensable for the development of CRC.

Furthermore, we found that the anti-tumorigenicity of *A.muciniphila* was weakened after AhR knockdown in the mouse models. Subsequently, gavage of *A. muciniphila* in ABX-treated AhR -deficient *Apc^Min/+^* mice did not reduce both tumor count and size significantly, further supporting the preventive and protective effects of *A. muciniphila*. Furthermore, results in the tumoroid tissues derived from the *Apc^Min/+^* mice and AhR-deleted mice implied that AhR is essential for the initiation, but not the maintenance of tumoroid changes.

The Wnt/β-catenin signaling pathway is known to be activated in colonic tumorigenesis with an increased proliferation of IECs 56. We observed that AhR play more important role in the proliferative capacity of colorectal cells than normal colon epithelial cells, indicating AhR initiates CSC self-renewal in a Wnt/β-catenin-dependent manner. One possibility is that function of AhR was now proved to regulate the development in CRC tumorigenesis. β-catenin interacts with AhR in the development of CRC, either directly or indirectly 57. Following IDO1 upregulation, β-catenin activation and subsequent AhR activation were observed in the mouse model 58. Our data are consistent with those in other studies indicating that AhR is an upstream of the Wnt/β-catenin pathway in CRC tumorigenesis, with a possible mechanism involving the phosphorylation of nuclear β-catenin and subsequent activation of TCF/LEF 24.

A low abundance of *A. muciniphila* is strongly associated with the development of CRC 59. However, *A. muciniphila* is rich in the feces of CRC patients with lower rate of progression of colon cancer 60. What can be understood that already in early stages of tumor development, the balance of gut microbiota is altered possibly contributing to the observed shift toward higher frequencies of AhR. What is more, Activation of the Wnt/β-catenin pathway induced by AhR is the initiating event in the majority of human colorectal cancers and one of the key regulators of CRC pathogenesis.

## Conclusions

**Figure 8:**
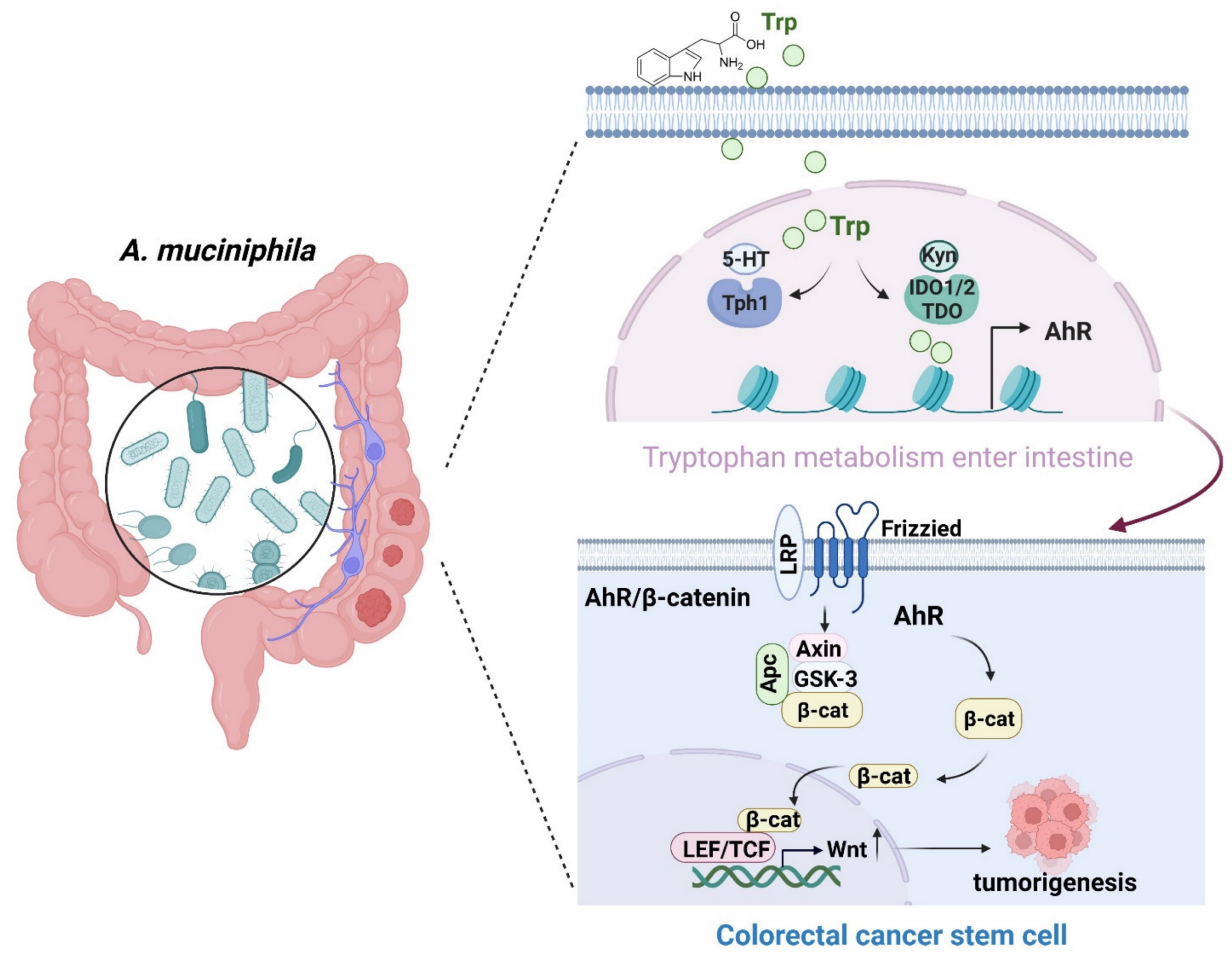
** Schematic diagram of A. muciniphila through Tryptophan metabolism mediated AhR/β-catenin pathway inhibit colorectal carcinogenesis.** Gut microbes synthesize Trp outside cells. Trp enters IECs through various amino acid transporters and is metabolized endogenously: TPH1 (or TPH2 in the periphery) is the rate-limiting enzyme in conversion to 5-HT, and IDO and TDO enzymes convert Trp into Kyn. Kyn functions as a ligand for the transcription factor AhR that translocates into the nucleus. AhR induced Wnt/β-catenin activation and transcription factor TCF/LEF activity and promotes IECs turn into tumorigenesis.

In summary, *A. muciniphila* might inhibit CRC progression via down-regulating AhR involved in the Wnt/β-catenin signaling pathway. *A. muciniphila* may be targeted to treat CRC (**Figure 8**), even though the underlying mechanisms need further study.

## Supplementary Material

Supplementary data, figures and tables.

## Abbreviations

*A. muciniphila*: *Akkermansia muciniphila*
CRC: colorectal cancer
A-CRA: advanced colorectal adenoma
AOM: azoxymethane
ABX: Antibiotics
DSS: Dextran sodium sulfate
KO: knockout
H&E: Hematoxylin-eosin staining
IHC: Immunohistochemistry
ACF: aberrant crypt foci
AhR: aryl hydrocarbon receptor
IDO1/2: indoleamine-2,3-dioxygenase 1/2
TDO2: tryptophan-2,3-dioxgenase
IECs: intestinal epithelia cells
ISCs: intestinal stem cells
Kyn: Kynurenine
KP: kynurenine pathway
qRT-PCR: Quantitative real-time polymerase chain reaction
