# Supplementary material for: Akkermansia muciniphila inhibits tryptophan metabolism via the AhR/β-catenin signaling pathway to counter the progression of colorectal cancer
Source: Int J Biol Sci. 2023 Aug 21;19(14):4393–410. doi: 10.7150/ijbs.85712 (PMC10535706; doi:10.7150/ijbs.85712)
Supplement: Supplementary file 1 — Supplementary data, figures and tables. [file ijbsv19p4393s1.pdf]

Supplementary Data

Electron microscopic

The intestinal tissue of AOM/DSS mice treatment with *A. muciniphila* (1×10^8 colony forming units) or Vehicle (E. coli MG1655 or the same volume of phosphate buffer saline) were excised and fixed in 0.1M phosphate buffer containing 2.5% glutaraldehyde and 2.0% paraformaldehyde (pH 7.4). Then the tissues were fixed, dehydrated, polymerized and then examined using the transmission electron microscope as previously described [1].

CRISPR/Cas9-mediated knockout of AhR

The genomic sequence of AhR was located at ensemble.org. Locate the exon or any exon of our interest (that may contain a functional domain, hot spot, etc.), copy and paste 23–500 nt (~200 nt optimal) onto crispr. mit.edu to design CRISPR sgRNA strands for nuckase. Pick the top-ranked strands (with predicted faithfulness scores close to 100), to ligate into CRISPR constructs, at least two for nuclease. For every sgRNA strand, two oligos were designed and ordered, forward and reverse, complimentary to each other, with 5' overhang CACC for one, AAAC for the other sticky ends for the BbsI/BsmBI site (**Figure 2E**). Perform the sub-cloning process: oligo insert annealing to form the oligo duplex, dilute the phosphorylated and annealed oligo duplexes 1:100 in H_2O, followed by the process of LentiCRISPR v2 plasmid digestion and oligo insert ligation. Each construct was quickly transformed into Stbl3-competent cells, and at least six clones of each construct were selected for Sanger sequencing to validate insert ligation using the U6-Forward primer.

Puromycin was used to weed out the CRISPR negative cells and the isogenic single-cell clones were obtained in a 96-well plate using serial dilution method. Single clones of transduced cells were screened for indels in AhR coding sequence by locus PCR/Sanger sequencing, and RT-PCR. Those without full-length AhR expression were used to perform further experiments.

Transfection of plasmids, siRNAs, and lentivirus production

Specific siRNAs were used to knock down AhR (Sequences of all the primers are shown in **Supplementary Table 1**; Qiagen). Transfection procedures were performed according to manufacturers' instructions, with Lipofectamin 2000 as transfection reagent (Invitrogen). Briefly, 2×10^4 cells were plated in each well of a 6-well plate and incubated overnight. A mixture of Lipofectamine 2000 (10 nM) with siRNA (50 nM) was added, followed by a 48 h incubation in regular

medium. sh- AhR and sh-control lentiviral particles used to transfect DLD-1 cells were generated by cotransfection of 293T cells. The GFP positive cells, transfected with sh-AhR-GFP-Lentivirus, were sorted and the stable clones were cultured as previously described [2].

Cell Culture and Reagents

Human colorectal cancer cell lines HCT-116, DLD-1, HT-29, LoVo, HCT-8, Caco-2, and NCM460 (human normal colonic epithelial) cells were purchased from the Shanghai Cell Collection (Shanghai, China). The NCM460, HCT-116, HCT-8, LoVo, HT-29, Caco-2, and SW480, SW620 cells were grown at 37 °C in a 5% CO_2 humidified atmosphere in RPMI 1640, F12K, DMEM and L-15 medium, respectively, supplemented with 10% (v/v) heat-inactivated fetal calf serum, 2 mM glutamine, 100 units/ml penicillin, and 100 μg/ml streptomycin (Invitrogen, Carlsbad, CA).

Animals and Xenograft Models

We have summarized all animal models in **Supplementary Table 7** and represented them with representative molding method etc. Male athymic nude mice (NCr-nu), 8-12 weeks old, were purchased from Sino-British SIPPR/BK Lab Animal Co., Ltd (Shanghai, China, license No. SCXK 2008-0016), and maintained under pathogen-free conditions. When the xenograft tumors reached an average size of 100 mm^3 (almost 8 days), all the animals were injected through the vena caudalis every 2 days as the above four groups mentioned. After treatment for 28 days, luciferase intensity was detected by bioluminescence. At the end point (when tumor volume reached ~2000 mm^3), the animals were euthanized; their tumor was excised, cleaned, and imaged; and their tumor mass was excised.

Tumor volume and two perpendicular diameters (A and B) were recorded every 3 days. The average tumor radius was calculated as (A+B)/4. Tumor volume (V) was estimated (assuming a spherical shape) using the formula $V = (4/3)\ \pi\ r^3$.

In vivo BrdU assay

According to 5-bromo-2-deoxyuridine (BrdU) in vivo kit's instructions (BrdU; Sigma-Aldrich), prepare a fresh BrdU solution at 10 mg/ml in saline every time and keep it refrigerated in the dark. The solution is sonicated in an ultrasound water bath for a few minutes immediately before injection. It was injected intraperitoneally into the mice once daily for 5 days as described previously [3], then intestine tissues were harvested after cleanout with PBS. The tissues were fixed on glass slides with 2.5% paraformaldehyde, Epitopes were retrieved by heat induction with Antigen Decloaker 10X

(Biocare Medical, Concord, CA) in a rice cooker for 10 minutes at 120°C. After blocking non-specific binding (Protein Block, 30 minutes, room temperature), tissues were incubated for 2 hrs at RT (room temperature) with mouse anti-BrdU (1:100, BD Biosciences, San Jose, CA). Then the tissues were labeled for 1 hr at RT with AlexaFluor-488 goat anti-mouse IgG (1:200), AlexaFluor-647 goat anti-chicken IgG (1:200). The cell nuclei were stained twice for 10 minutes at RT with DAPI as a counter stain. Images were takenby the Leica DMi8 Laser Scanning Confocal.

Supplementary Table

Table 1. PCR primers

gene	Forward primer	Reverse primer
A. muciniphila	CAGCACGTGAAGGTGGGGAC	CCTTGCGGTTGGCTTCAG
AhR	ACATCACCTACGCCAGTCG	CGCTTGGAAGGATTTGACTTGA
β-catenin	AACAGGGTCTGGGACATTAGTC	CGAAAGCCAATCAAACACAAAC
GSK-3β	CAGACCTGCCTTACGACTATGG	CTCGGTGGCGTTGAGATTGTT
Axin2	GACGGACAGCAGTGTAGATG	GGGTTCTCGGGAAATGA
c-Myc	CGAGCAAGGACGCGACTCTC	GAGGCTGCTGGTTTTCCACTAC
Cyclin D1	TGTCGTTCGAACCCCTCAAG	TTGCAGTAACTCGTCGGGTC
Sox2	CTTGAGAGAAAAAGGAGAAC	CACACTAAATATACCCACTGG
OCT-4	CCTTGAAGGGGAAGTAGGAC	CAAGGCCTCCGTGCTATATCC
Nanog	GGTTGAAGACTAGCAATGGTCTGA	TGCAATGGATGCTGGGATACTC
GAPDH	TGTGTCCGTCGTGGATCTGA	CCTGCTTCACCACCTTCTTGA

Table 2. Information on patients

Characteristics	Polyp patients(N=46)	A-CRA patients(N=38)	CRC patients(N=42)
Gender			
Male	22	20	23
Female	24	18	19
Age/year			
>60	8	11	24
≤60	38	27	18
Mean ± SD	43±7	55±8	59±6
TNM staging			
I	-	-	18
II	-	-	11
III	-	-	13
IV	-	-	0
Nodal status			
Positive	-	-	26
Negative	-	-	16

Table 3. Comparison of the abundances of *A. muciniphila* in human example (%)

Group	n	The abundances of *A. muciniphila*	
		Low	High
CAC (Mild Hyperplasia)	46	14(30%)	32(70%)
A-CRA (Moderate and Severe Hyperplasia)	38	22(57.89%)	16(42.11%)
CRC	42	28(66.67%)	14(33.33%)

Polyps with subtle hyperplastic features were classified as colonic mucosa with polypoid hyperplasia (CMPH) and those without histologic abnormalities as normal mucosa. [4]

Table 4. Tumor weight and tumor inhibition rate in mouse xenografts model

Group	Tumor weight	Tumor inhibition rate	P value
DLD-1	2.14+0.18	-	-
DLD-1/AhR$^{-/-}$	1.83+0.25	14.49%	0.047

Inhibition rate (%)=(average weight of Control group-average weight of Treatment group)/ average weight of Control group×100

Table 5. DAI score in AOM/DSS mice (at the end of experiment)

Group	Symptom score			Disease activity index (DAI)
	weight loss	stool consistency	occult blood	
WT	1.6	2.4	3.2	2.4
AhR$^{-/-}$Cre	1.4	2	3.2	2.2

Symptom score including weight loss score, stool consistency score and occult blood score. The evaluation criterion of Symptom score was described as previous [1].

DAI=(weight loss score+ stool consistency score+ occult blood score)/3

Table 6. Histopathologic analysis of neoplastic lesions and the degree of dysplasia

Group	carcinoma/tumor number	malignant degree of carcinoma
Apc$^{Min/+}$	7/20	35%
Apc$^{Min/+}$+ *A. muciniphila*	5/20	25%
Apc$^{Min/+}$ AhR$^{-/-}$	4/20	20%
Apc$^{Min/+}$ AhR$^{-/-}$+ *A. muciniphila*	5/20	25%

Histopathologic analysis of neoplastic lesions and the degree of dysplasia were assessed according to standard criteria and classification of adenomas of the colon. tubular adenoma with high-grade dysplasia characterized; low grade adenocarcinomas with focal submucosal invasion [5].

Table 7. Animal model in the experiment

Naming	Abbreviation	Graphic	Method of establishing animal model
AOM/DSS-induced CRC mice	AOM/DSS	no graphic in this article	on day 1, the mice were injected with AOM (12.5 mg/kg, i.p.). After 1 week, the mice were given drinking water containing 2.5% DSS for 7 days, followed by tap water for 14 days for recovery. This cycle was repeated twice.
Apc$^{Min/+}$ C57BL/6J	*Apc*$^{Min/+}$	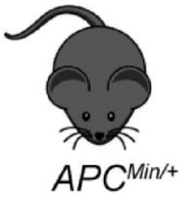	From Jackson Laboratory
C57BL/6-Ahr^{em1Stck}/J	AhR$^{fl/fl}$	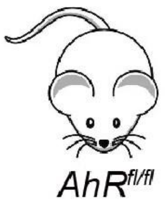	From Jackson Laboratory

B6.Cg-Tg(Vil1-cre)997Gum/J	Villin-Cre^{+}	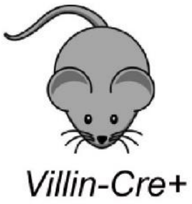	From Jackson Laboratory
AhR$^{-/-}$Cre		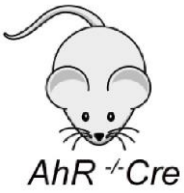	crossed mice with a floxed Ahr locus to Villin-Cre mice to restrict Ahr deficiency to IECs.
Apc$^{Min/+}$ AhR $^{-/-}$	*Apc*$^{Min/+}$ AhR $^{-/-}$	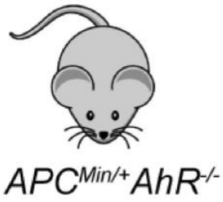	crossed mice with a floxed AhR$^{-/-}$Cre locus to *Apc*$^{Min/+}$ mice to restrict Ahr deficiency to *Apc*$^{Min/+}$ mice.
DLD-1/AhR$^{+/+}$	DLD-1/AhR	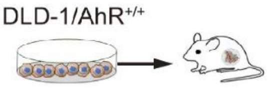	Male athymic nude mice (NCr-nu), 8-12 weeks old, were subcutaneously inoculated with DLD-1 cells.
DLD-1/AhR$^{-/-}$		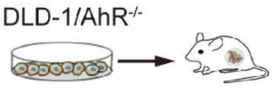	Male athymic nude mice (NCr-nu), 8-12 weeks old, were subcutaneously inoculated with DLD-1/AhR$^{-/-}$ cells.

Supplementary Figure

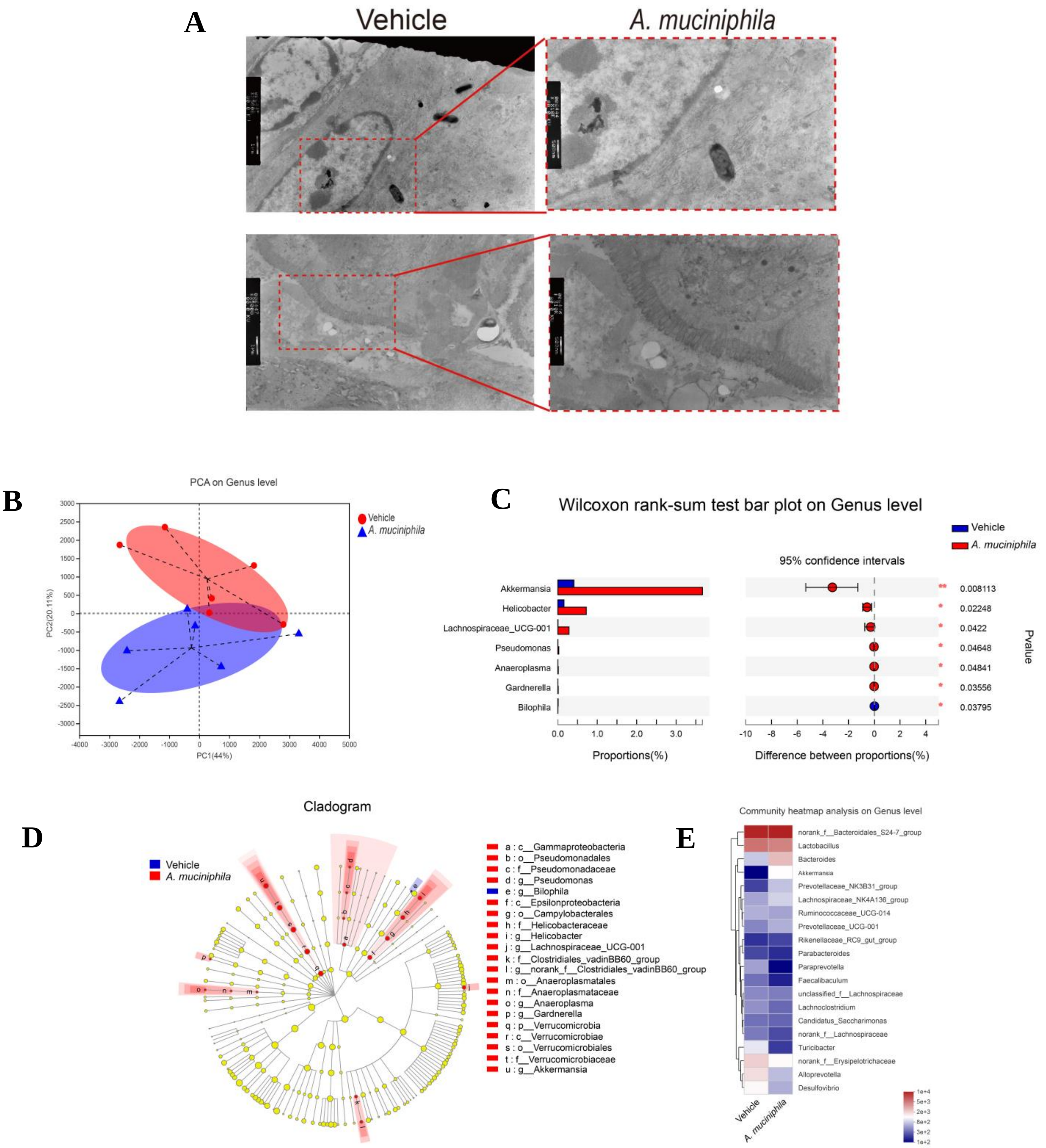

Supplementary Fig. 1. Effects of *A. muciniphila* on tumor growth and the abundance of microbiota in mice.

(**A**) Electron microscopy in the lumen infiltration of pseudo-GF/AOM/DSS mice after infected with *A. muciniphila* for 12 weeks. Both microvilli and goblet cells can also be seen. Magnification bars, left: 1μM; right: 500 nM. (**B**) Principle component analysis (PCA) analysis at the genus-level, which was

used to study the differences in the composition of bacterial communities in the fecal samples between pseudo-GF/AOM/DSS mice infected with *A. muciniphila* and Vehicle group. (**C**) Bar plot of compositional differences at the genus level in the gut microbiome of mice in the combination GF/AOM/DSS mice infected with *A. muciniphila* and Vehicle group by the Wilcoxon rank-sum test. Data are expressed as mean ± SEM. * $0.01 < P \leq 0.05$, ** $0.001 < P \leq 0.01$, *** $P \leq 0.001$, Two-sided Hypotheses. (**D**) Cladogram generated from the linear discriminant analysis effect size (LEfSe) between pseudo-GF/AOM/DSS mice infected with *A. muciniphila* and Vehicle group. The analyses were performed at the end of the experiment. (**E**) Heat map of Genus with relative abundances that are significantly different from their relative abundances at the time of *A. muciniphila* administration. The differentially enriched bacterial Genus in pseudo-GF/AOM/DSS mice receiving Vehicle or *A. muciniphila*. The relative abundance between control and treatment mice for the genus was calculated for each time.

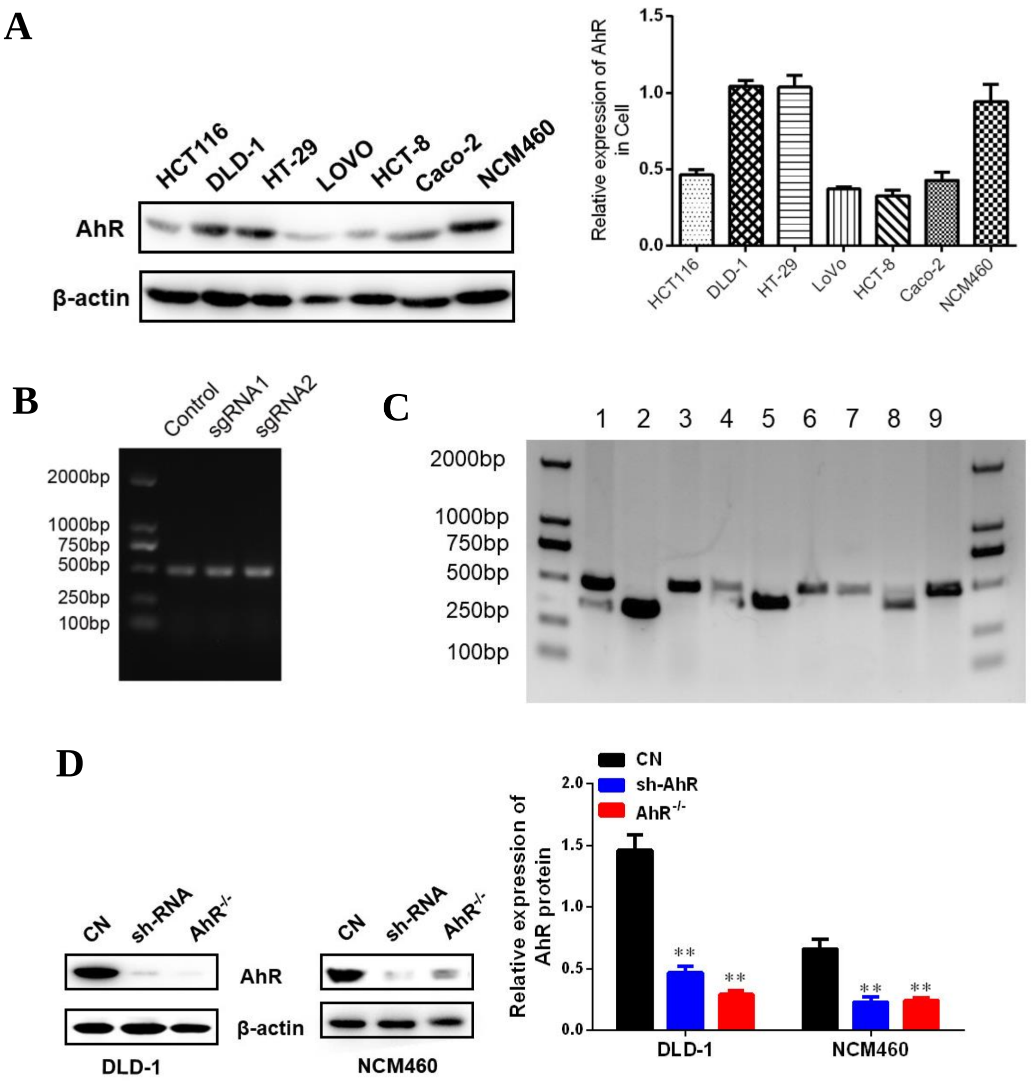

E

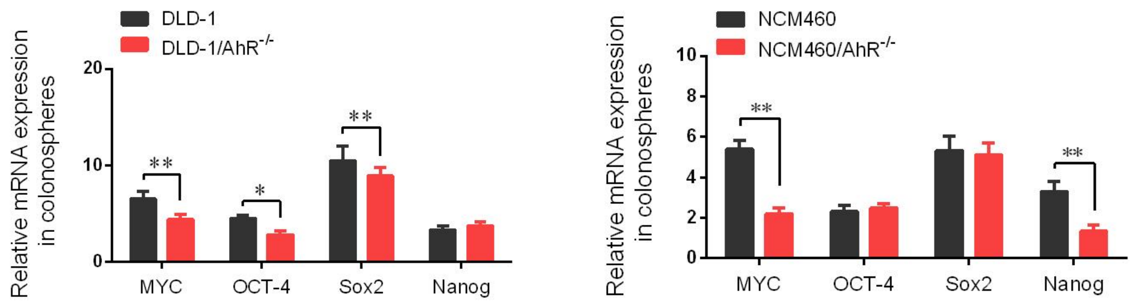

Supplementary Fig. 2. CRISPR/Cas9-mediated AhR knockout

(**A**) Expression level of AhR in several different CRC cell lines. (**B**) Validation of CRISPR knockout in single-cell clones by PCR and DNA sequencing. (**C**) Northern blot analysis of AhR expression. Twenty micrograms of total RNA from AhR knockout ($AhR^{-/-}$) cells (2 and 5) and wild-type cells were run in agarose/formaldehyde gels. (**D**) Expression level of AhR in different treatment with DLD-1 cells and NCM460 cells. Data from triplicate experiments are presented as mean ± SD. $^{**}P < 0.01$ *vs*. control. (**E**) mRNA Expression levels of MYC, Sox2, OCT-4, and Nanog in DLD-1 and NCM460 cells with AhR knockout or not, were analyzed through quantitative real-time PCR.

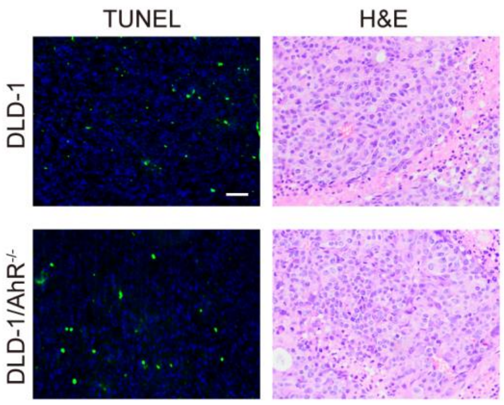

Supplementary Figure 3 In vivo antitumor activity of *A. muciniphila*.

H&E and TUNEL staining of tumors between DLD-1/$AhR^{-/-}$ tumour-bearing mice and DLD-1 tumour-bearing mice, scale bar, 100 μm.

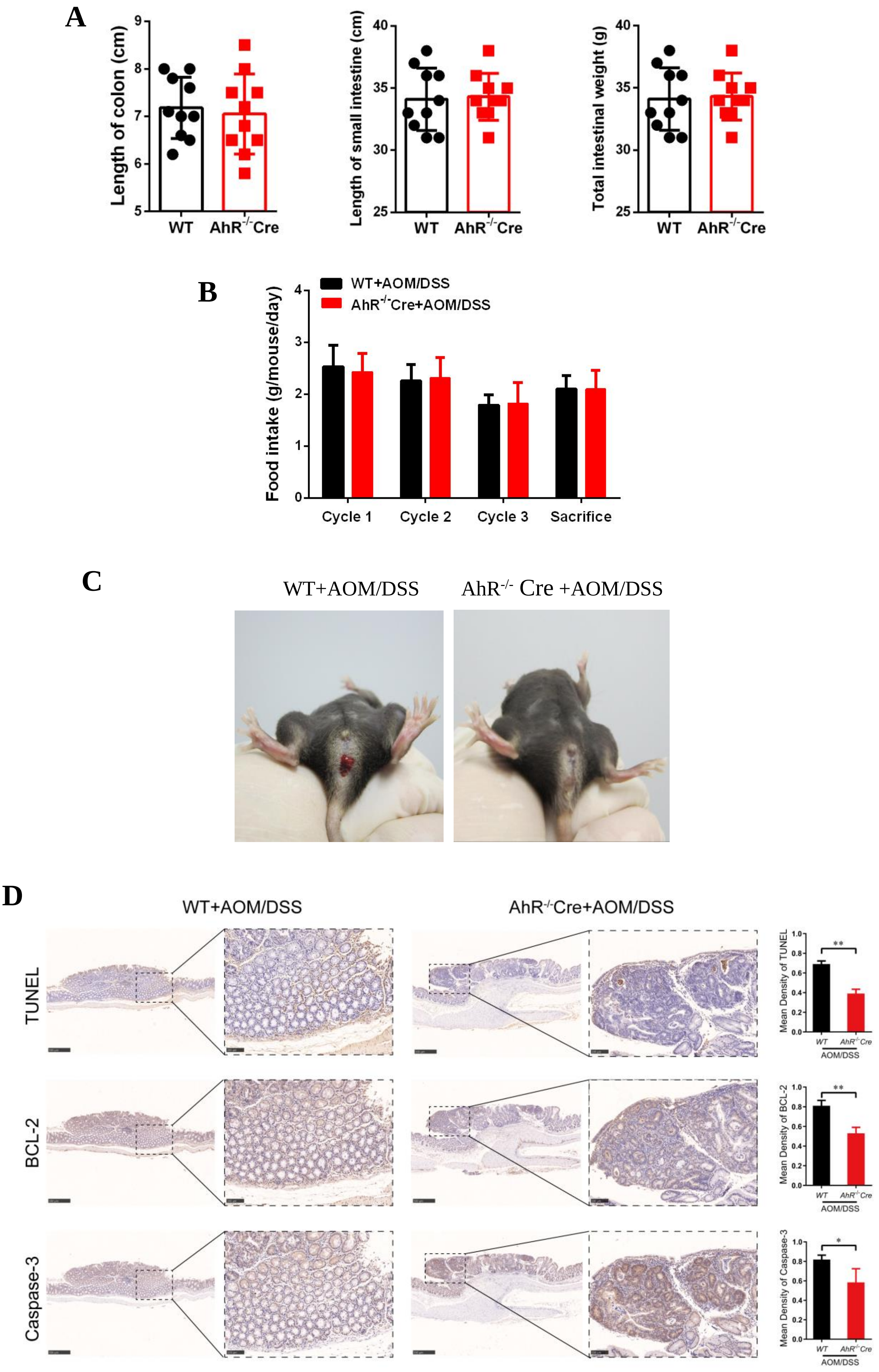

A
Length of colon (cm)
WT
AhR-/-Cre
Length of small intestine (cm)
Total intestinal weight (g)
B
WT+AOM/DSS
AhR-/-Cre+AOM/DSS
Food intake (g/mouse/day)
Cycle 1
Cycle 2
Cycle 3
Sacrifice
C
WT+AOM/DSS
AhR-/- Cre +AOM/DSS
D
WT+AOM/DSS
AhR-/-Cre+AOM/DSS
TUNEL
BCL-2
Caspase-3
Mean Density of TUNEL
Mean Density of BCL-2
Mean Density of Caspase-3
AOM/DSS

Supplementary Fig. 4. The effect of AhR deficiency in AOM/DSS mice model

(**A**) Effects of AhR deficiency on colon length (Left), small intestine length (Middle) and total intestine weight between WT and AhR$^{-/-}$ Cre mice. (**B**) Food intake in two groups. (**C**) The mice are from AOM/DSS model. Signs of illness were monitored daily and body weight was recorded every other day. Bloody stools were observed and photoed during the whole stage of the experiment. (**D**) Immunohistochemical staining using an antibody against TUNEL, BCL-2, and Caspase-3 in the colons of WT and AhR$^{-/-}$ Cre mice. Scale bars, 100 μM. Data of eight mice per experimental group are shown as means ± SD, with Welch's correction through one-tailed t-test. *P < 0.05, **P < 0.01 *vs*. WT.

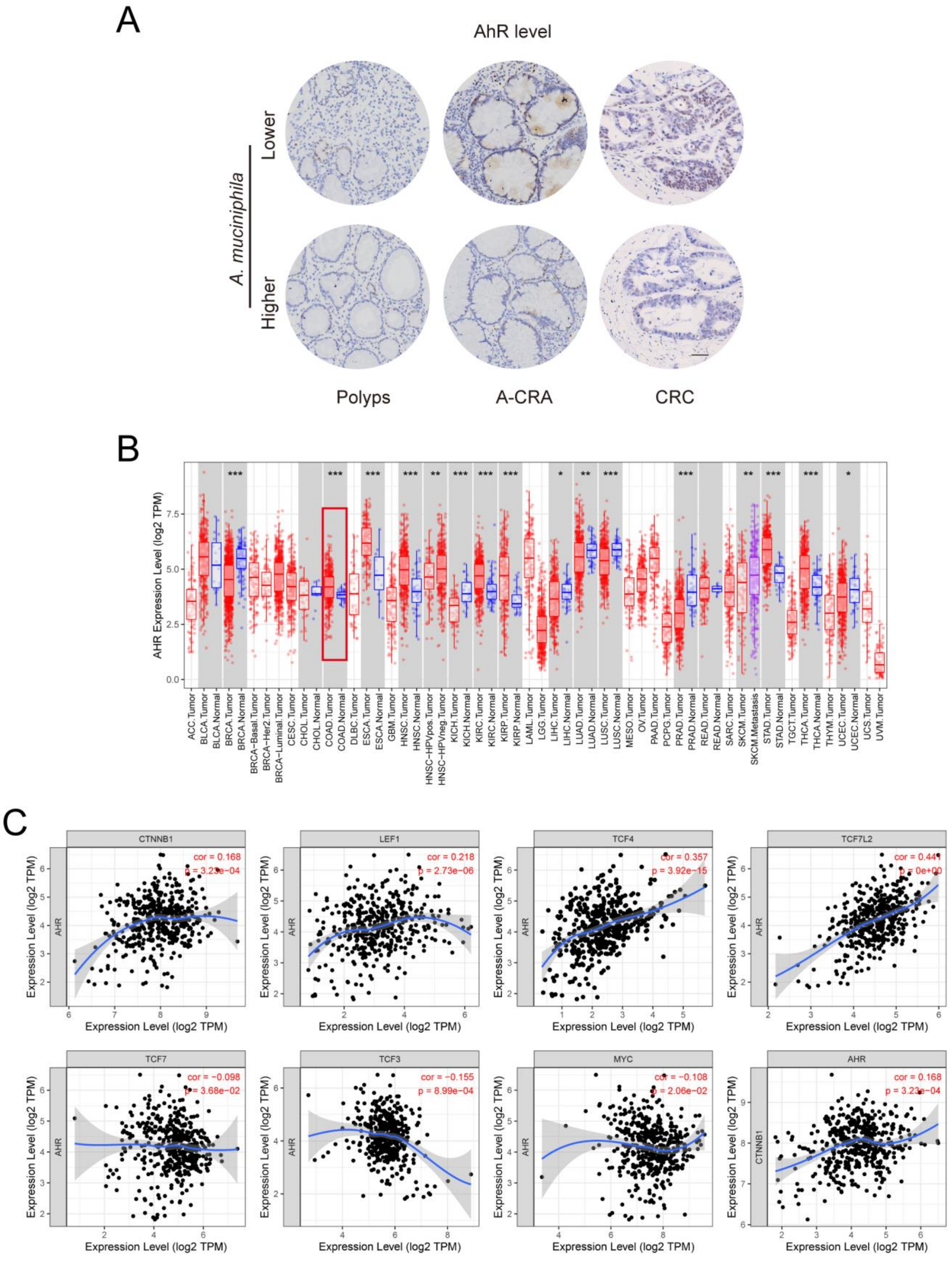

Supplementary Figure 5 The expression of AhR on patients with *A. muciniphila* higher and lower.

(**A**) AhR expression analysis in CAC, A-CRA, and CRC with *A. muciniphila* abundance is markedly

decreased as Fig. 1A. scale bar, 50 μm. (**B**) AhR expression levels in different tumor types from TCGA database were determined by TIMER (**P* < 0.05, ***P* <0.01, ****P* < 0.001). The expression of AhR was significantly higher in COAD Tumor (red) than that in COAD Normal group (blue); *P* < 0.001. (**C**) Correlation analysis between the relative mRNAs of AhR and some typical target genes of Wnt/β-catenin pathway.

Supplemental references

1. Sui H, Zhang L, Gu K, Chai N, Ji Q, Zhou L, Wang Y, Ren J, Yang L, Zhang B, Hu J, Li Q. YYFZBJS ameliorates colon cancer progression in ApcMin/+ mice by remodeling gut microbiota and inhibiting regulatory T-cell generation. Cell Commun Signal. 2020;18(1):113.

2. Sui H, Cai GX, Pan SF, Deng WL, Wang YW, Chen ZS, Cai SJ, Zhu HR, Li Q. miR-200c attenuates P-gp mediated MDR and metastasis by targeting JNK2/c-Jun signaling pathway in colorectal cancer. Mol Cancer Ther, 2014, 13(12): 3137-3151 5.

3. Fu T, Coulter S, Yoshihara E, Oh TG, Fang S, Cayabyab F, Zhu Q, Zhang T, Leblanc M, Liu S, He M, Waizenegger W, Gasser E, Schnabl B, Atkins AR, Yu RT, Knight R, Liddle C, Downes M, Evans RM. FXR Regulates Intestinal Cancer Stem Cell Proliferation. Cell. 2019; 176(5):1098-1112.

4. Hissong E, Fernandes H, Jessurun J. Colonic Mucosa With Polypoid Hyperplasia. Am J Clin Pathol. 2019;152(4):423-430.

5. Poutahidis T, Rao VP, Olipitz W, Taylor CL, Jackson EA, Levkovich T, Lee CW, Fox JG, Ge Z, Erdman SE. CD4+ lymphocytes modulate prostate cancer progression in mice. Int J Cancer. 2009;125(4):868-78.
